# Analysis of Drug-Induced Gastrointestinal Obstruction and Perforation Using the Japanese Adverse Drug Event Report Database

**DOI:** 10.3389/fphar.2021.692292

**Published:** 2021-07-26

**Authors:** Riko Satake, Kiyoka Matsumoto, Mizuki Tanaka, Ririka Mukai, Kazuyo Shimada, Yu Yoshida, Misaki Inoue, Shiori Hasegawa, Kazuhiro Iguchi, Hiroaki Ikesue, Shinya Shimizu, Shohei Nishida, Akio Suzuki, Tohru Hashida, Mitsuhiro Nakamura

**Affiliations:** ^1^ Laboratory of Drug Informatics, Gifu Pharmaceutical University, Gifu, Japan; ^2^ Department of Pharmacy, Kobe City Medical Center General Hospital, Kobe, Japan; ^3^ Laboratory of Community Pharmacy, Gifu Pharmaceutical University, Gifu, Japan; ^4^ Department of Pharmacy, Gifu University Hospital, Gifu, Japan

**Keywords:** The Japanese Adverse Drug Event Report, gastrointestinal obstruction, gastrointestinal perforation, ileus, pharmacovigilance, time-to-onset profile

## Abstract

Drug-induced gastrointestinal obstruction (DIGO) and gastrointestinal perforation (DIGP) may be the result of gastrointestinal hypomotility and severe constipation, which may lead to potentially fatal complications of bowel ischemia, sepsis and perforation. We evaluated the onset profile of DIGs (DIGO and DIGP) associated with prescription drugs by analyzing data in the Japanese Adverse Drug Event Report (JADER) database. We selected 161 DIG-related drugs and categorized them into 19 classes based on the Anatomical Therapeutic Chemical (ATC) Classification System. Finally, we focused on 58 drugs and conducted subsequent analyses for the time-to-onset and outcomes. We extracted 79 preferred terms (PTs) with the strings “ileus,” “stenosis,” “obstruction,” “obstructive,” “impaction,” “perforation,” “perforated,” “hypomotility,” and “intussusception” from the Standardized Medical Dictionary for Regulatory Activities (MedDRA) Queries (SMQs) of SMQ20000104: gastrointestinal perforation, ulcer, hemorrhage, obstruction non-specific findings/procedures; SMQ20000105: gastrointestinal obstruction; and SMQ20000107: gastrointestinal perforation. Among the 667, 729 reports in the JADER database submitted between April 2004 and November 2020, we identified 11,351 occurrences of DIGs. The reporting odds ratios (RORs) (95% confidence interval) of “barium sulfate containing X-ray media,” “drugs for treatment of hyperkalemia and hyperphosphatemia,” and “oral bowel cleanser” were 142.0 (127.1–158.6), 25.8 (23.1–28.8), and 29.7 (24.8–35.6), respectively. The median number of days (interquartile range) until the onset of an adverse event caused by each drug category was as follows: barium sulfate containing X-ray contrast media [2.0 (1.0–3.0)], diazepines, oxazepines, thiazepines, and oxepines [8.0 (8.0–18.5)], drugs for treatment of hyperkalemia and hyperphosphatemia [29.0 (8.0–55.0)], non-selective monoamine reuptake inhibitors [19.0 (7.0–47.5)], and oral bowel cleanser [0.0 (0.0–0.0)]. Depending on the drug, the time to onset of side effects ranged from days to several months. Our results highlighted the need to perform detailed monitoring of each drug for possible association with DIGs, which might otherwise have fatal consequences.

## Introduction

Drug-induced gastrointestinal obstruction (DIGO) and gastrointestinal perforation (DIGP) may be the result of gastrointestinal hypomotility and severe constipation, which may lead to potentially fatal complications of bowel ischemia, sepsis and perforation. (Keller and Layer, 2009; Nielsen and Meyer, 2012). DIGs may also result from mucosal damage caused by NSAIDs (even COX inhibitors) or by cytotoxic antineoplastic agents, such as vinca alkaloids or topoisomerase 1 (TOP1) inhibitors (Bjarnason et al., 2018). It is important for clinicians to know the timing and outcome profile of DIGs (DIGO, DIGP, and ileus) (Ministry of Health, Labour and Welfare, 2008). Although information on DIG risk has accumulated through several clinical trials, it does not reflect the complexities of real-life practice. Several drugs, such as α-glucosidase inhibitors, antineoplastic agents, antipsychotics, dantrolene, drugs for urinary frequency and incontinence, opium alkaloids, and polystyrene sulfonate are known to be associated with paralytic ileus (Ministry of Health, Labour and Welfare, 2008). Severe paralytic ileus is relatively uncommon, occurring at a frequency of 6% of the total number of paralytic ileus cases (Ministry of Health, Labour and Welfare, 2008). The prognosis of ileus associated with α-glucosidase inhibitors is often reported to be good (Oba et al., 2001). On the contrary, it is reported that constipation is associated with a greater number of clozapine (an atypical antipsychotic drug)-related deaths than agranulocytosis (Every-Palmer et al., 2017). Oral bowel cleansers and sodium polystyrene sulfonate have more severe outcomes (Ajinomoto Pharma Co Ltd, 2003; Noel et al., 2019). Healthcare professionals should be aware of the potential risks of DIGs. The detailed time-to-onset profiles of DIGs in clinical settings are not clear for many drugs.

In Japan, adverse events (AEs) during the post-marketing phase are reported and managed by the Pharmaceuticals and Medical Devices Agency (PMDA). The Japanese Adverse Drug Event Report (JADER) database is a spontaneous reporting system (SRS) of the real-world data voluntarily submitted by healthcare professionals, pharmaceutical companies, and patients (van Puijenbroek et al., 2002; Hasegawa et al., 2020). The JADER database is publicly available on the PMDA website (http://www.pmda.go.jp) and is used in pharmacovigilance assessments. The reporting odds ratio (ROR) is a pharmacovigilance index that has been developed for drug-associated AEs (van Puijenbroek et al., 2002). Furthermore, time-to-onset analysis using the Weibull shape parameter (WSP) is a useful tool for AE signal detection (Sauzet et al., 2013; Hasegawa et al., 2020).

There are several good commercial SRS database analysis services. However, the data cleaning process is often a black box for users. It is difficult for such commercial services to support the complex and flexible analyses required by researchers, based on the stratified AE group, the stratified drug efficacy group, age, polypharmacy, and detailed patient background. For this purpose, the DIG onset profile of multiple drugs is accessible from the JADER database. In the present study, we evaluated the onset profile of DIGs associated with prescription drugs by analyzing the data present in the JADER database. We assessed DIGs by determining the RORs and time-to-onset analysis. To the best of our knowledge, this is the first study to evaluate the association of prescription drugs with DIGs.

## Methods

### Data Source

The JADER data from April 2004 to November 2020 were obtained from the PMDA website. All data in the JADER database were fully anonymized by the regulatory authority of Japan: PMDA. The JADER database consists of four tables: 1) DEMO (patient’s information, such as age, sex, and reporting year); 2) DRUG (drug name, route, and start and end date of drug administration); 3) HIST (primary illness); and 4) REAC (AEs, outcome, and onset date). Outcomes are classified as “death,” “with sequelae,” “not recovered,” “convalescent,” “recovery,” and “others.” We integrated a relational database using the four tables with the FileMaker Pro 14 software (FileMaker, Inc, Santa Clara, CA, United States). Drugs in JADER were assigned to three categories, namely “suspected,” “concomitant,” and “interacting,” drugs, according to the anticipated degree of involvement in AEs. Only reports with the drug code “suspected” were included in this analysis.

### Drug Selection

A number of drugs are known to produce various patterns of DIGs (Ministry of Health, Labour and Welfare, 2008). The World Health Organization Collaborating Center for Drug Statistics Methodology (www.whocc.no/atc_ddd_index/) described the Anatomical Therapeutic Chemical (ATC) Classification System. In this study, we first listed the drug names and efficacy groups related to DIGs described in the guidelines and previous studies (Ministry of Health, Labour and Welfare, 2005). Second, we selected the ATC classification to which the drug and drug efficacy belong, and examined the reporting status of the JADER database for all the drugs contained in it. Third, we selected 161 DIG-related drugs and categorized them into 19 ATC-drug classes (Supplementary Table S1). Fifty-eight drugs were reported to cause DIGs (Supplementary Table S1). Finally, we focused on those 58 drugs and conducted subsequent analyses for the time-to-onset and outcomes. There are 1054 DIG-related drugs in the JADER database, and in this study we did not analyze the drugs that were not included in the 19 ATC classes that we focused on. Indeed, it is possible to enumerate the unknown ROR signals for all drugs, but this was not done in the present study. Because celecoxib is listed in ATC classifications of other antineoplastic agents (L01XX) and coxibs (M01AH) and opium is listed in antipropulsives (A07DA) and natural opium alkaloids (N02AA), the two drugs are listed as-is in Table 1 and Supplementary Table S1. Oral bowel cleanser was not listed in the ATC Classification System.

**TABLE 1:** Number of reports and reporting odds ratio of drug-induced gastrointestinal obstruction and perforation.

Drugs	Total (n)	Total (79 PTs)		Obstruction (23PTs)		Perforation (34PTs)		Ileus (8PTs)		SMQ: 2000104 (76 PTs)		SMQ: 20000105 (79 PTs)		SMQ: 20000107 (96 PTs)	
		Case (n)	ROR^a^ (95% CI^b^)	Case (n)	ROR^a^ (95% CI^b^)	Case (n)	ROR^a^ (95% CI^b^)	Case (n)	ROR^a^ (95% CI^b^)	Case (n)	ROR^a^ (95% CI^b^)	Case (n)	ROR^a^ (95% CI^b^)	Case (n)	ROR^a^ (95% CI^b^)
	667729	11351		1428		6212		3030		3838		5693		8110	
Alpha glucosidase inhibitors (ATC^c^ code: A10BF)	1966	99	3.1 (2.5–3.8)^d^	47	11.8 (8.8–15.8)^d^	4	0.2 (0.1–0.6)	49	5.7 (4.3–7.6)^d^	350	41.1 (36.5–46.4)^d^	96	6.1 (4.9–7.4)^d^	15	0.6 (0.4–1.0)
acarbose	366	19	3.2 (2.0–5.0)^d^	9	11.8 (6.1–23.0)^d^	2	0.6 (0.1–2.3)	8	4.9 (2.4–9.9)^d^	54	30.4 (22.7–40.6)^d^	17	5.7 (3.5–9.2)^d^	2	0.4 (0.1–1.8)
miglitol	443	38	5.4 (3.9–7.6)^d^	14	15.4 (9.0–26.2)^d^	1	-	22	11.5 (7.5–17.7)^d^	40	17.3 (12.5–24.0)^d^	38	11.0 (7.9–15.3)^d^	2	0.4 (0.1–1.5)
voglibose	1157	42	2.2 (1.6–3.0)^d^	24	10.0 (6.7–15.1)^d^	1	-	19	3.7 (2.3–5.8)^d^	256	52.6 (45.6–60.7)^d^	41	4.3 (3.1–5.9)^d^	11	0.8 (0.4–1.4)
Antipropulsives (ATC^c^ code: A07DA)	194	29	10.2 (6.9–15.1)^d^	4	9.8 (3.7–26.5)^d^	5	2.8 (1.2–6.9)^d^	20	25.4 (16.0–40.4)^d^	13	12.5 (7.1–21.9)^d^	24	16.5 (10.7–25.3)^d^	7	3.0 (1.4–6.5)^d^
opium	13	1	-	0	-	1	-	0	-	2	31.5 (7.0–142.0)^d^	0		2	14.8 (3.3–66.7)^d^
loperamide	181	28	10.6 (7.1–15.9)^d^	4	10.6 (3.9–28.5)^d^	4	2.4 (0.9–6.5)	20	27.4 (17.2–43.7)^d^	11	11.2 (6.1–20.7)^d^	24	17.8 (11.6–27.4)^d^	5	2.3 (0.9–5.6)
Barium sulfate containing X-ray contrast media (ATC^c^ code: V08BA)	1512	1044	142.0 (127.1–158.6)^d^	95	33.4 (27.0–41.4)^d^	937	204.2 (183.4–227.3)^d^	25	3.7 (2.5–5.5)^d^	6	0.7 (0.3–1.5)	133	11.5 (9.6–13.7)^d^	974	167.2 (150.1–186.2)^d^
barium sulfate with suspending agents	1512	1044	142.0 (127.1–158.6)^d^	95	33.4 (27.0–41.4)^d^	937	204.2 (183.4–227.3)^d^	25	3.7 (2.5–5.5)^d^	6	0.7 (0.3–1.5)	133	11.5 (9.6–13.7)^d^	974	167.2 (150.1–186.2)^d^
Butyrophenone derivatives (ATC^c^ code: N05AD)	2141	57	1.6 (1.2–2.1)^d^	8	1.8 (0.9–3.5)	0	-	48	5.1 (3.8–6.8)^d^	11	0.9 (0.5–1.6)	60	3.4 (2.6–4.4)^d^	2	0.1 (0.0–0.3)
haloperidol	1816	46	1.5 (1.1–2.0)^d^	8	2.1 (1.03–4.2)^d^	0	-	37	4.6 (3.3–6.4)^d^	11	1.1 (0.6–1.9)	46	3.0 (2.3–4.1)^d^	1	-
bromperidol	169	7	2.5 (1.2–5.3)^d^	0	-	0	-	7	9.5 (4.5–20.3)^d^	0	-	10	7.3 (3.9–13.9)^d^	1	-
droperidol	139	4	1.7 (0.6–4.6)	0	-	0	-	4	6.5 (2.4–17.6)^d^	0	-	4	3.4 (1.3–9.3)^d^	0	-
Coxibs (ATC^c^ code: M01AH)	3851	63	1.0 (0.7–1.2)	8	1.0 (0.5–1.9)	49	1.4 (1.04–1.8)^d^	5	0.3 (0.1–0.7)	7	0.3 (0.1–0.7)	15	0.5 (0.3–0.8)	51	1.1 (0.8–1.4)
celecoxib	3851	63	1.0 (0.7–1.2)	8	1.0 (0.5–1.9)	49	1.4 (1.04–1.8)^d^	5	0.3 (0.1–0.7)	7	0.3 (0.1–0.7)	15	0.5 (0.3–0.8)	51	1.1 (0.8–1.4)
Dantrolene and derivatives (ATC^c^ code: M03CA)	215	9	2.5 (1.3–4.9)^d^	3	6.6 (2.1–20.7)^d^	0	-	6	6.3 (2.8–14.2)^d^	1	-	9	5.1 (2.6–9.9)^d^	0	-
dantrolene	215	9	2.5 (1.3–4.9)^d^	3	6.6 (2.1–20.7)^d^	0	-	6	6.3 (2.8–14.2)^d^	1	-	9	5.1 (2.6–9.9)^d^	0	-
Diazepines, oxazepines, thiazepines and oxepines (ATC^c^ code: N05AH)	8065	384	3.0 (2.7–3.3)^d^	37	2.2 (1.6–3.0)^d^	30	0.4 (0.3–0.6)	331	10.4 (9.3–11.7)^d^	58	1.3 (0.97–1.6)	373	6.0 (5.4–6.6)^d^	44	0.4 (0.3–0.6)
clozapine	2282	256	7.5 (6.5–8.5)^d^	22	4.6 (3.0–7.0)^d^	30	1.4 (0.99–2.0)	219	25.0 (21.7–28.9)^d^	23	1.8 (1.2–2.7)^d^	239	14.2 (12.3–16.2)^d^	44	1.6 (1.2–2.2)^d^
olanzapine	2556	62	1.4 (1.1–1.9)^d^	9	1.7 (0.9–3.2)	0	-	53	4.7 (3.6–6.2)^d^	8	0.5 (0.3–1.1)	65	3.1 (2.4–3.9)^d^	0	-
quetiapine	2870	59	1.2 (0.9–1.6)	4	0.7 (0.2–1.7)	0	-	54	4.3 (3.3–5.6)^d^	27	1.6 (1.1–2.4)^d^	62	2.6 (2.0–3.3)^d^	0	-
asenapine	357	7	1.2 (0.5–2.4)	2	2.6 (0.7–10.6)	0	-	5	3.1 (1.3–7.5)^d^	0	-	7	2.3 (1.1–4.9)^d^	0	-
Drugs for treatment of hyperkalemia and hyperphosphatemia (ATC^c^ code: V03AE)	1544	463	25.8 (23.1–28.8)^d^	53	17.2 (13.0–22.7)^d^	332	30.8 (27.2–34.8)^d^	88	13.6 (11.0–16.9)^d^	17	1.9 (1.2–3.1)^d^	148	12.6 (10.6–15.0)^d^	356	25.4 (22.6–28.7)^d^
polystyrene sulfonate	444	199	47.8 (39.6–57.7)^d^	15	16.5 (9.8–27.6)^d^	171	68.6 (56.5–83.1)^d^	15	7.7 (4.6–12.9)^d^	8	3.2 (1.6–6.4)^d^	39	11.3 (8.1–15.7)^d^	179	56.2 (46.4–68.0)^d^
sevelamer	406	111	22.0 (17.6–27.3)^d^	12	14.3 (8.0–25.5)^d^	82	27.3 (21.4–34.8)^d^	18	10.2 (6.4–16.4)^d^	0	-	30	9.3 (6.4–13.5)^d^	86	22.1 (17.4–28.0)^d^
lanthanum carbonate	512	148	23.8 (19.6–28.8)^d^	26	25.4 (17.1–37.8)^d^	76	18.8 (14.7–24.0)^d^	53	25.8 (19.3–34.3)^d^	8	2.7 (1.4–5.5)^d^	77	20.9 (16.3–26.6)^d^	86	16.6 (13.1–20.9)^d^
colestilan	56	1	-	0	-	1	-	0	-	0	-	0		1	-
ferric citrate	126	4	1.9 (0.7–5.1)	0	-	2	1.7 (0.4–6.9)	2	3.5 (0.9–14.3)	1	-	2	1.9 (0.5–7.6)	4	2.7 (0.99–7.2)
Drugs for urinary frequency and incontinence (ATC^c^ code: G04BD)	3766	96	1.5 (1.2–1.9)^d^	38	4.9 (3.5–6.7)^d^	3	0.1 (0.0–0.3)	54	3.2 (2.5–4.2)^d^	22	1.0 (0.7–1.5)	92	2.9 (2.4–3.6)^d^	4	0.1 (0.0–0.2)
flavoxate	138	2	0.9 (0.2–3.4)	2	6.9 (1.7–27.8)^d^	0	-	0	-	1	-	2	1.7 (0.4–6.9)	0	-
oxybutynin	70	7	6.4 (2.9–14.0)^d^	2	13.7 (3.4–56.1)^d^	0	-	5	16.9 (6.8–42.0)^d^	2	5.1 (1.2–20.8)^d^	7	12.9 (5.9–28.3)^d^	0	-
propiverine	295	21	4.4 (2.8–6.9)^d^	10	16.5 (8.8–31.0)^d^	0	-	11	8.5 (4.7–15.6)^d^	2	1.2 (0.3–4.7)	20	8.5 (5.4–13.4)^d^	0	-
tolterodine	120	6	3.0 (1.3–6.9)^d^	4	16.1 (5.9–43.8)^d^	0	-	2	3.7 (0.9–15.1)	1	-	6	6.1 (2.7–13.9)^d^	0	-
solifenacin	865	27	1.9 (1.3–2.7)^d^	8	4.4 (2.2–8.8)^d^	0	-	19	5.0 (3.1–7.8)^d^	6	1.2 (0.5–2.7)	28	3.9 (2.7–5.7)^d^	1	-
fesoterodine	1240	17	0.8 (0.5–1.3)	6	2.3 (1.02–5.1)^d^	1	-	9	1.6 (0.8–3.1)	3	0.4 (0.1–1.3)	16	1.5 (0.9–2.5)	1	-
mirabegron	1037	16	0.9 (0.6–1.5)	6	2.7 (1.2–6.1)^d^	2	0.2 (0.1–0.8)	8	1.7 (0.9–3.4)	7	1.2 (0.6–2.5)	13	1.5 (0.9–2.6)	2	0.2 (0.0–0.6)
Natural opium alkaloids (ATC^c^ code: N02AA)	1901	134	4.4 (3.7–5.3)^d^	19	4.8 (3.0–7.5)^d^	29	1.7 (1.1–2.4)^d^	83	10.3 (8.2–12.8)^d^	20	1.8 (1.2–2.9)^d^	111	7.3 (6.0–8.9)^d^	35	1.5 (1.1–2.1)^d^
morphine	738	39	3.2 (2.3–4.5)^d^	7	4.5 (2.1–9.5)^d^	11	1.6 (0.9–2.9)	21	6.5 (4.2–10.0)^d^	7	1.7 (0.8–3.5)	32	5.3 (3.7–7.6)^d^	13	1.5 (0.8–2.5)
opium	13	1	-	0	-	1	-	0	-	2	31.5 (7.0–142.0)^d^	0		2	14.8 (3.3–66.7)^d^
oxycodone	988	94	6.1 (4.9–7.6)^d^	12	5.8 (3.3–10.2)^d^	17	1.9 (1.2–3.0)^d^	62	15.0 (11.5–19.4)^d^	11	2.0 (1.1–3.5)^d^	79	10.2 (8.1–12.9)^d^	20	1.7 (1.1–2.6)^d^
Non-selective monoamine reuptake inhibitors (ATC^c^ code: N06AA)	1995	26	0.8 (0.5–1.1)	2	0.5 (0.1–1.9)	2	0.1 (0.0–0.4)	21	2.3 (1.5–3.6)^d^	7	0.6 (0.3–1.3)	23	1.4 (0.9–2.0)	2	0.1 (0.0–0.3)
imipramine	300	2	0.4 (0.1–1.6)	0	-	0	-	2	1.5 (0.4–5.9)	2	1.2 (0.3–4.7)	2	0.8 (0.2–3.1)	0	-
clomipramine	402	5	0.7 (0.3–1.8)	2	2.3 (0.6–9.4)	1	-	2	1.1 (0.3–4.4)	0	-	4	1.2 (0.4–3.1)	1	-
amitriptyline	409	8	1.2 (0.6–2.3)	0	-	1	-	6	3.3 (1.5–7.3)^d^	3	1.3 (0.4–4.0)	6	1.7 (0.8–3.9)	1	-
nortriptyline	52	1	-	0	-	0	-	1	-	0	-	1		0	-
amoxapine	506	7	0.8 (0.4–1.7)	0	-	0	-	7	3.1 (1.5–6.5)^d^	1	-	7	1.6 (0.8–3.4)	0	-
maprotiline	299	3	0.6 (0.2–1.8)	0	-	0	-	3	2.2 (0.7–6.9)	1	-	3	1.2 (0.4–3.7)	0	-
Oral bowel cleanser	527	177	29.7 (24.8–35.6)^d^	76	83.0 (64.7–106.4)^d^	65	15.1 (11.7–19.6)^d^	38	17.3 (12.4–24.0)^d^	0	-	119	34.6 (28.2–42.5)^d^	70	12.6 (9.8–16.2)^d^
sodium potassium combination (including sodium potassium ascorbic acid combination agent)	527	177	29.7 (24.8–35.6)^d^	76	83.0 (64.7–106.4)^d^	65	15.1 (11.7–19.6)^d^	38	17.3 (12.4–24.0)^d^	0	-	119	34.6 (28.2–42.5)^d^	70	12.6 (9.8–16.2)^d^
Other antineoplastic agents (ATC^c^ code: L01XX)	8201	101	0.7 (0.6–0.9)	12	0.7 (0.4–1.2)	72	0.9 (0.7–1.2)	15	0.4 (0.2–0.7)	16	0.3 (0.2–0.5)	31	0.4 (0.3–0.6)	82	0.8 (0.7–1.0)
asparaginase	932	9	0.6 (0.3–1.1)	0	-	4	0.5 (0.2–1.2)	4	0.9 (0.4–2.5)	2	0.4 (0.1–1.5)	5	0.6 (0.3–1.5)	5	0.4 (0.2–1.1)
hydroxycarbamide	385	2	0.3 (0.1–1.2)	0	-	1	-	1	-	1	-	1		3	0.6 (0.2–2.0)
estramustine	274	1	-	0	-	0	-	1	-	2	1.3 (0.3–5.1)	1		0	-
celecoxib	3851	63	1.0 (0.7–1.2)	8	1.0 (0.5–1.9)	49	1.4 (1.04–1.8)^d^	5	0.3 (0.1–0.7)	7	0.3 (0.1–0.7)	15	0.5 (0.3–0.8)	51	1.1 (0.8–1.4)
anagrelide	277	1	-	0	-	1	-	0	-	1	-	0		1	-
eribulin	1041	4	0.2 (0.1–0.6)	0	-	2	0.2 (0.1–0.8)	2	0.4 (0.1–1.7)	2	0.3 (0.1–1.3)	2	0.2 (0.1–0.9)	2	0.2 (0.0–0.6)
aflibercept	1295	21	1.0 (0.6–1.5)	4	1.4 (0.5–3.9)	15	1.2 (0.8–2.1)	2	0.3 (0.1–1.4)	1	-	7	0.6 (0.3–1.3)	20	1.3 (0.8–2.0)
Other antipsychotics (ATC^c^ code: N05AX)	9313	197	1.3 (1.1–1.4)^d^	31	1.6 (1.1–2.2)^d^	9	0.1 (0.1–0.2)	159	4.0 (3.4–4.7)^d^	61	1.1 (0.9–1.5)	195	2.5 (2.2–2.9)^d^	10	0.1 (0.0–0.2)
risperidone	3825	75	1.2 (0.9–1.5)	16	2.0 (1.2–3.2)^d^	3	0.1 (0.0–0.3)	56	3.3 (2.5–4.3)^d^	27	1.2 (0.8–1.8)	77	2.4 (1.9–3.0)^d^	4	0.1 (0.0–0.2)
mosapramine	25	5	14.5 (5.4–38.5)^d^	0	-	0	-	5	54.9 (20.6–146.5)^d^	0	-	5	29.1 (10.9–77.6)^d^	0	-
zotepine	445	30	4.2 (2.9–6.1)^d^	6	6.4 (2.9–14.3)^d^	0	-	25	13.2 (8.8–19.7)^d^	8	3.2 (1.6–6.4)^d^	30	8.4 (5.8–12.2)^d^	0	-
aripiprazole	3393	62	1.1 (0.8–1.4)	6	0.8 (0.4–1.8)	5	0.2 (0.1–0.4)	52	3.5 (2.6–4.6)^d^	24	1.2 (0.8–1.8)	59	2.1 (1.6–2.7)^d^	5	0.1 (0.0–0.3)
paliperidone	1360	22	1.0 (0.6–1.4)	3	1.0 (0.3–3.2)	1	-	18	3.0 (1.9–4.7)^d^	2	0.3 (0.1–1.0)	21	1.8 (1.2–2.8)^d^	1	-
brexpiprazole	265	3	0.7 (0.2–2.1)	0	-	0	-	3	2.5 (0.8–7.8)	0	-	3	1.3 (0.4–4.2)	0	-
Phenothiazines with aliphatic side-chain (ATC^c^ code: N05AA)	2075	78	2.3 (1.8–2.8)^d^	9	2.0 (1.1–3.9)^d^	1	-	70	7.8 (6.1–9.9)^d^	9	0.8 (0.4–1.5)	77	4.5 (3.6–5.7)^d^	1	-
chlorpromazine	951	35	2.2 (1.6–3.1)^d^	7	3.5 (1.6–7.3)^d^	1	-	28	6.7 (4.6–9.8)^d^	3	0.5 (0.2–1.7)	33	4.2 (3.0–5.9)^d^	1	-
levomepromazine	1124	43	2.3 (1.7–3.1)^d^	2	0.8 (0.2–3.3)	0	-	42	8.6 (6.3–11.8)^d^	6	0.9 (0.4–2.1)	44	4.8 (3.5–6.5)^d^	0	-
Poly (ADP-ribose) polymerase (PARP) inhibitors (ATC^c^ code: L01XK)	860	19	1.3 (0.8–2.1)	6	3.3 (1.5–7.4)^d^	2	0.2 (0.1–1.0)	11	2.8 (1.6–5.2)^d^	0	-	17	2.4 (1.5–3.8)^d^	2	0.2 (0.0–0.8)
olaparib	859	19	1.3 (0.8–2.1)	6	3.3 (1.5–7.4)^d^	2	0.2 (0.1–1.0)	11	2.9 (1.6–5.2)^d^	0	-	17	2.4 (1.5–3.8)^d^	2	0.2 (0.0–0.8)
Proteasome inhibitors (ATC^c^ code: L01XG)	3929	142	2.2 (1.8–2.6)^d^	11	1.3 (0.7–2.4)	19	0.5 (0.3–0.8)	111	6.6 (5.4–8.0)^d^	18	0.8 (0.5–1.3)	125	3.9 (3.2–4.7)^d^	27	0.6 (0.4–0.8)
bortezomib	1043	126	8.0 (6.7–9.7)^d^	7	3.2 (1.5–6.7)^d^	17	1.8 (1.1–2.9)^d^	101	24.3 (19.7–29.9)^d^	13	2.2 (1.3–3.8)^d^	110	14.0 (11.4–17.0)^d^	25	2.0 (1.3–3.0)^d^
ixazomib	2886	16	0.3 (0.2–0.5)	4	0.6 (0.2–1.7)	2	0.1 (0.0–0.3)	10	0.8 (0.4–1.4)	5	0.3 (0.1–0.7)	15	0.6 (0.4–1.0)	2	0.1 (0.0–-.2)
Topoisomerase 1 (TOP1) inhibitors (ATC^c^ code: L01CE)	6436	297	2.8 (2.5–3.2)^d^	41	3.1 (2.2–4.2)^d^	169	2.9 (2.5–3.4)^d^	82	2.9 (2.3–3.6)^d^	78	2.1 (1.7–2.7)^d^	133	2.5 (2.1–3.0)^d^	213	2.8 (2.5–3.3)^d^
irinotecan	6436	297	2.8 (2.5–3.2)^d^	41	3.1 (2.2–4.2)^d^	169	2.9 (2.5–3.4)^d^	82	2.9 (2.3–3.6)^d^	78	2.1 (1.7–2.7)^d^	133	2.5 (2.1–3.0)^d^	213	2.8 (2.5–3.3)^d^
Vinca alkaloids and analogues (ATC^c^ code: L01CA)	5062	202	2.4 (2.1–2.8)^d^	19	1.8 (1.1–2.8)^d^	80	1.7 (1.4–2.1)^d^	77	3.5 (2.7–4.3)^d^	25	0.9 (0.6–1.3)	131	3.1 (2.6–3.7)^d^	103	1.7 (1.4–2.1)^d^
vinblastine	447	5	0.7 (0.3–1.6)	0	-	0	-	5	2.5 (1.03–6.0)^d^	1	0.4 (0.1–2.8)	6	1.6 (0.7–3.5)	0	-
vincristine	3546	168	2.9 (2.5–3.4)^d^	16	2.1 (1.3–3.5)^d^	72	2.2 (1.8–2.8)^d^	54	3.4 (2.6–4.5)^d^	23	1.1 (0.7–1.7)	104	3.6 (2.9–4.3)^d^	93	2.2 (1.8–2.7)^d^
vindesine	235	5	1.3 (0.5–3.0)	0	-	2	0.9 (0.2–3.7)	3	2.8 (0.9–8.9)	1	0.7 (0.1–5.3)	3	1.5 (0.5–4.7)	2	0.7 (0.2–2.8)
vinorelbine	834	24	1.7 (1.1–2.6)^d^	3	1.7 (0.5–5.2)	6	0.8 (0.3–1.7)	15	4.0 (2.4–6.7)^d^	0	-	18	2.6 (1.6–4.1)^d^	8	0.8 (0.4–1.6)

a ROR: Reporting odds ratio

b CI: Confidence interval

c ATC: the Anatomical Therapeutic Chemical (ATC) classification system described by the World Health Organization Collaborating Centre for Drug Statistics Methodology (www.whocc.no/atc_ddd_index)

d Lower limit of 95% CI > 1 Number of cases <2

### Definition of AEs

**FIGURE 1:**
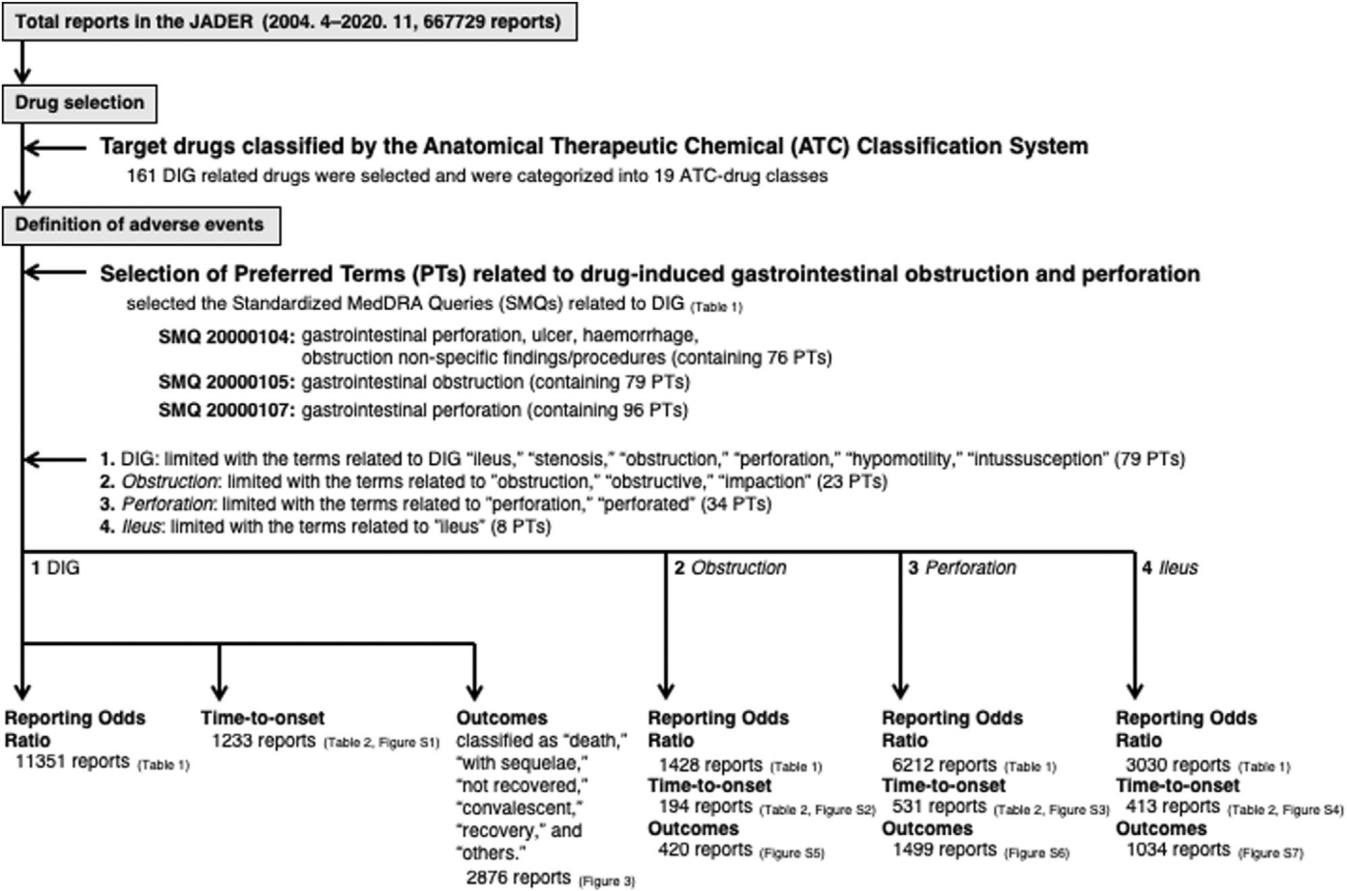
Flowchart of data analysis.

AEs were coded with terms in the Medical Dictionary for Regulatory Activities (MedDRA, https://www.meddra.org), which is the terminology dictionary used in the JADER database (The International Council for Harmonization of Technical Requirements for Pharmaceuticals for Human Use [ICH], Introductory Guide MedDRA Version 23.1). The AEs in this study relied on the definitions provided by the MedDRA, ver. 23.1 (MedDRA MSSO, 2020). The Standardized MedDRA Queries (SMQs), which are predefined sets of MedDRA terms aimed at useful data retrieval and the presentation of relevant individual case pharmacovigilance topics were built by the Maintenance and Support Services Organization. SMQs are groupings of PTs according to the level that relates to a defined medical condition, and the included terms may relate to signs, symptoms, diagnoses, syndromes, physical findings, laboratory, and other physiological test data, among others (MedDRA MSSO, 2020). The grouping of SMQs exists in both “narrow” and “broad” scope. We could not find a gold standard for the selection of PTs of DIGs. We selected PTs in this study based on the “narrow” scope for SMQ20000104: gastrointestinal perforation, ulcer, hemorrhage, obstruction non-specific findings/procedures (containing 76 PTs); SMQ20000105: gastrointestinal obstruction (containing 79 PTs); and SMQ20000107: gastrointestinal perforation (containing 96 PTs) (Figure 1, Supplementary Table S2). The specificity of the “narrow” terms for each SMQ was considered to be low in the specificity required for this study. Therefore, to allow for the identification of cases that are highly likely to represent DIGs, we extracted 79 preferred terms (PTs) with the strings “ileus,” “stenosis,” “obstruction,” “obstructive,” “impaction,” “perforation,” “perforated,” “hypomotility,” and “intussusception” from SMQ20000104: gastrointestinal perforation, ulcer, hemorrhage, obstruction non-specific findings/procedures; SMQ20000105: gastrointestinal obstruction; and SMQ20000107: gastrointestinal perforation (Figure 1; Table 1, Supplementary Table S2). Furthermore, we limited the number of strings for extraction for more specific evaluation. Then, we extracted 23 specific PTs for *obstruction* with the strings “obstruction,” “obstructive,” and “impaction” from SMQ20000104, SMQ20000105, and SMQ20000107 (Supplementary Table S2). We extracted 34 specific PTs for *perforation* with the strings “perforation” and “perforated” from SMQ20000104, SMQ20000105, and SMQ20000107 (Supplementary Table S2). We extracted eight specific PTs for *ileus* with the string “ileus” from SMQ20000104, SMQ20000105, and SMQ20000107 (Supplementary Table S2).

### Signal Detection

**FIGURE 2:**
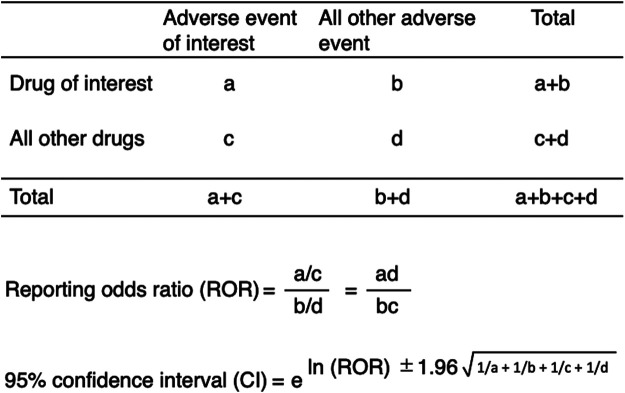
Two by two contingency table for analysis.

We calculated ROR, which is the authorized pharmacovigilance index to analyze the association between drugs and DIGs using a two-by-two contingency (Poluzzi et al., 2012) (Figure 2). All the reported AEs of interest, such as DIGs, were defined as cases and all other reported AEs were defined as non-cases. The number of co-occurrences of interest was defined as “a.” The number of co-occurrences with a drug of interest, but without an AE of interest, was defined as “b.” Those without a drug of interest, but with an AE of interest, were defined as “c.” The number of co-occurrences without either a drug or AE of interest was defined as “d.” We calculated the RORs as (a:c)/(b:d) (Figure 2) for the cases and non-cases. RORs were expressed as point estimates with 95% CIs. Signals were considered statistically significant when the lower limit of the 95% CI was above 1. At least two cases were required to define the signal (Poluzzi et al., 2012).

### Time-To-Onset Analysis

Time-to-onset was calculated as the time elapsed between the patient’s last prescription and the occurrence of the AE. The analysis period was 90 days after the first prescription date. The median duration, quartiles, and WSPs were used to evaluate the time-to-onset data (Sauzet et al., 2013; Hasegawa et al., 2020). The scale parameter *α* of the Weibull distribution determines the scale of the distribution function. A larger scale value (*α*) stretches the data distribution, whereas a smaller scale value shrinks it. The shape parameter, *β*, of the Weibull distribution determines the shape of the distribution function. A larger value of *β* gives a left-skewed curve, whereas a smaller value gives a right-skewed curve. The WSP *β* was used to indicate the hazard without a reference population. If *β* is equal to 1, the hazard is estimated to be constant over time. When *β* is greater than one and the 95% CI of *β* exceeds 1, the hazard is considered to increase over time.

### Evaluation of Outcomes

A mosaic plot of the contingency table was constructed with the drug or age category (X) and the outcome category (Y). The proportions on the *x*-axis represent the number of observations for each level of the X variable. The mosaic plot is divided into rectangles, and the vertical length of each rectangle is proportional to the size of the Y variable at each level of the X variable.

## Results

The JADER database contains 667,729 reports submitted between April 2004 and November 2020 (Figure 1). We identified 11,351 occurrences of DIGs in these reports. The number of reports on “ileus,” “intestinal obstruction,” “ileus paralytic,” “intestinal perforation,” “mechanical ileus,” “subileus,” “postoperative ileus,” “procedural intestinal perforation,” “ileus spastic,” and “gallstone ileus” were 1939, 1,206, 762, 668, 193, 136, 26, 6, 2, and 1, respectively (Supplementary Table S2). The RORs of 58 drugs are summarized in Table 1 according to Supplementary Table S1. The ROR [95% confidence interval (CI)] of “antipropulsives,” “barium sulfate containing X-ray media,” “drugs for treatment of hyperkalemia and hyperphosphatemia,” and “oral bowel cleanser” were 10.2 (6.9–15.1), 142.0 (127.1–158.6), 25.8 (23.1–28.8), and, 29.7 (24.8–35.6), respectively (Table 1).

For the time-to-onset analysis, we extracted combinations that had complete information for the start date of drug administration and the date of AE onset. We evaluated 18 classifications of drugs (Table 2, Supplementary Figure S1). The median number of days (interquartile range) until AE onset caused by each drug category was as follows: α-glucosidase inhibitors, 16.5 (3.3–48.5); antipropulsives, 7.0 (3.0–11.0); barium sulfate containing X-ray contrast media, 2.0 (1.0–3.0); butyrophenone derivatives, 7.0 (4.0–7.0); coxibs, 26.5 (12.0–52.8); diazepines, oxazepines, thiazepines, and oxepines, 8.0 (8.0–18.5); drugs for treatment of hyperkalemia and hyperphosphatemia, 29.0 (8.0–55.0); drugs for urinary and incontinence, 13.0 (7.5–32.0); natural opium alkaloids, 12.0 (6.0–32.0); non-selective monoamine reuptake inhibitors, 19.0 (7.0–47.5); oral bowel cleansers, 0.0 (0.0–0.0); other antineoplastic agents, 16.5 (8.3–46.3); other antipsychotics, 8.0 (2.0–15.0); phenothiazines with aliphatic side-chain, 8.0 (4.0–8.0); poly (ADP-ribose) polymerase (PARP) inhibitors, 16.5 (11.0–38.5); proteasome inhibitors, 13.0 (6.0–30.5); TOP1 inhibitors, 19.0 (8.0–39.5); and vinca alkaloids and analogues, 8.0 (4.0–20.0). The lower limits of the 95% CIs of the WSP *β* value of antipropulsives, barium sulfate containing X-ray contrast media, non-selective monoamine reuptake inhibitors, oral bowel cleanser, and TOP1 inhibitors were >1.

**TABLE 2:** Medians and weibull parameter of each drugs.

Drugs	Total (79 PTs)				Obstruction (23PTs)				Perforation (34 PTs)				Ileus (8 PTs)			
	Case (n)	Median (quartile)	Scale palameter (95% CI^a^)	Shape palameter (95% CI^a^)	Case (n)	Median (quartile)	Scale palameter (95% CI^a^)	Shape palameter (95% CI^a^)	Case (n)	Median (quartile)	Scale palameter (95% CI^a^)	Shape palameter (95% CI^a^)	Case (n)	Median (quartile)	Scale palameter (95% CI^a^)	Shape palameter (95% CI^a^)
Alpha glucosidase inhibitors (ATC^b^ code: A10BF)	24	16.5 (3.3–48.5)	25.4 (15.1–41.3)^c^	0.9 (0.6–1.2)	9	15.0 (2.0–42.5)	32.9 (12.6–81.5)^c^	1.1 (0.5–2.0)	1	4.0 (4.0–4.0)	-	-	14	23.0 (3.8–51.0)	31.3 (16.9–55.8)^c^	1.1 (0.6–1.6)
acarbose	5	15.0 (5.0–74.5)	36.5 (7.1–174.6)^c^	0.9 (0.3–1.8)	3	15.0 (4.0–83.0)	48.0 (5.5–469.2)^c^	1.2 (0.3–3.3)	0	-	-	-	2	36.0 (6.0–66.0)	-	-
miglitol	13	17.0 (3.5–38.0)	23.1 (11.2–45.4)^c^	0.9 (0.6–1.4)	3	16.0 (2.0–47.0)	33.5 (9.3–129.2)^c^	2.1 (0.4–5.5)	1	4.0 (4.0–4.0)	-	-	9	22.0 (3.5–41.5)	28.4 (12.8–60.0)^c^	1.1 (0.6–1.8)
voglibose	6	20.0 (1.5–49.3)	26.8 (6.6–100.2)^c^	0.9 (0.4–1.8)	3	2.0 (2.0–38.0)	-	-	0	-	-	-	3	49.0 (0.0–50.0)	49.7 (48.7–50.9)^c^	118.8 (26.0–320.6)^c^
Antipropulsives (ATC^b^ code: A07DA)	15	7.0 (3.0–11.0)	10.6 (7.1–15.2)^c^	1.6 (1.03–2.4)^c^	1	3.0 (3.0–3.0)	-	-	1	0.0 (0.0–0.0)	-	-	13	7.0 (5.0–13.5)	11.2 (7.6–16.0)^c^	1.8 (1.1–2.6)^c^
opium	0	-	-	-	0	-	-	-	0	-	-	-	0	-	-	-
loperamide	15	7.0 (3.0–11.0)	10.6 (7.1–15.2)^c^	1.6 (1.03–2.4)^c^	1	3.0 (3.0–3.0)	-	-	1	0.0 (0.0–0.0)	-	-	13	7.0 (5.0–13.5)	11.2 (7.6–16.0)^c^	1.8 (1.1–2.6)^c^
Barium sulfate containing X-ray contrast media (ATC^b^ code: V08BA)	346	2.0 (1.0–3.0)	2.7 (2.6–2.9)^c^	1.7 (1.6–1.8)^c^	41	2.0 (0.5–5.0)	3.7 (3.0–4.5)^c^	1.9 (1.4–2.5)^c^	302	2.0 (1.0–3.0)	2.6 (2.4–2.8)^c^	1.7 (1.6–1.8)^c^	7	3.0 (2.0–5.0)	2.3 (1.2–4.2)^c^	2.2 (0.8–4.5)
barium sulfate with suspending agents	346	2.0 (1.0–3.0)	2.7 (2.6–2.9)^c^	1.7 (1.6–1.8)^c^	41	2.0 (0.5–5.0)	3.7 (3.0–4.5)^c^	1.9 (1.4–2.5)^c^	302	2.0 (1.0–3.0)	2.6 (2.4–2.8)^c^	1.7 (1.6–1.8)^c^	7	3.0 (2.0–5.0)	2.3 (1.2–4.2)^c^	2.2 (0.8–4.5)
Butyrophenone derivatives (ATC^b^ code: N05AD)	19	7.0 (4.0–7.0)	9.5 (5.7–15.4)^c^	1.0 (0.7–1.4)	2	4.0 (1.0–7.0)	-	-	0	-	-	-	18	7.0 (6.3–7.0)	10.2 (6.2–16.5)^c^	1.1 (0.8–1.4)
haloperidol	17	7.0 (5.5–7.0)	7.5 (5.7–9.8)^c^	2.0 (1.3–2.8)^c^	2	4.0 (1.0–7.0)	-	-	0	-	-	-	16	7.0 (7.0–7.0)	7.9 (6.2–10.1)^c^	2.3 (1.5–3.1)^c^
bromperidol	1	59.0 (59.0–59.0)	-	-	0	-	-	-	0	-	-	-	1	59.0 (59.0–59.0)	-	-
droperidol	1	2.0 (2.0–2.0)	-	-	0	-	-	-	0	-	-	-	1	2.0 (2.0–2.0)	-	-
Coxibs (ATC^b^ code: M01AH)	18	26.5 (12.0–52.8)	33.5 (22.4–48.8)^c^	1.4 (0.9–2.0)	2	12.0 (12.0–12.0)	-	-	16	29.5 (13.8–56.3)	37.0 (24.9–53.6)^c^	1.5 (0.9–2.2)	0	-	-	-
celecoxib	18	26.5 (12.0–52.8)	33.5 (22.4–48.8)^c^	1.4 (0.9–2.0)	2	12.0 (12.0–12.0)	-	-	16	29.5 (13.8–56.3)	37.0 (24.9–53.6)^c^	1.5 (0.9–2.2)	0	-	-	-
Dantrolene and derivatives (ATC^b^ code: M03CA)	0	-	-	-	0	-	-	-	0	-	-	-	0	-	-	-
dantrolene	0	-	-	-	0	-	-	-	0	-	-	-	0	-	-	-
Diazepines, oxazepines, thiazepines and oxepines (ATC^b^ code: N05AH)	53	8.0 (8.0–18.5)	18.9 (14.3–24.8)^c^	1.1 (0.9–1.3)	6	7.5 (5.5–45.3)	16.8 (3.9–66.6)^c^	0.8 (0.4–1.5)	2	32.0 (25.0–39.0)	-	-	36	8.0 (8.0–19.3)	20.6 (14.3–29.2)^c^	1.0 (0.8–1.3)
clozapine	15	20.0 (9.0–39.0)	29.7 (21.1–40.9)^c^	1.8 (1.1–2.7)^c^	2	27.5 (6.0–49.0)	-	-	2	32.0 (25.0–39.0)	-	-	22	22.0 (9.8–39.8)	31.1 (23.3–40.8)^c^	1.7 (1.2–2.4)^c^
olanzapine	19	8.0 (7.0–8.0)	15.8 (9.0–27.0)^c^	0.9 (0.7–1.2)	2	7.5 (7.0–8.0)	-	-	0	-	-	-	19	8.0 (8.0–8.0)	18.7 (10.4–32.6)^c^	0.9 (0.6–1.2)
quetiapine	16	8.0 (8.0–8.0)	8.6 (4.4–16.6)^c^	0.8 (0.6–1.1)	2	6.0 (4.0–8.0)	-	-	0	-	-	-	15	8.0 (8.0–8.0)	3.6 (1.3–9.6)^c^	0.6 (0.4–0.8)
asenapine	3	8.0 (5.0–44.0)	20.4 (1.2–395.9)^c^	0.9 (0.2–2.5)	1	44.0 (44.0–44.0)	-	-	0	-	-	-	2	6.5 (5.0–8.0)		
Drugs for treatment of hyperkalemia and hyperphosphatemia (ATC^b^ code: V03AE)	84	29.0 (8.0–55.0)	36.1 (29.0–44.5)^c^	1.1 (0.9–1.3)	7	39.0 (3.0-58.0)	38.3 (12.3–111.5)^c^	1.0 (0.4–1.8)	51	34.0 (12.0–55.0)	40.2 (31.9–50.3)^c^	1.3 (1.03–1.6)^c^	27	13.0 (3.0–52.0)	29.2 (18.0–46.0)^c^	0.9 (0.6–1.2)
polystyrene sulfonate	35	15.0 (6.0–52.0)	26.7 (18.6–37.5)^c^	1.0 (0.8–1.4)	2	45.0 (1.0–89.0)	-	-	30	19.0 (11.0–52.0)	30.5 (22.9–40.1)^c^	1.4 (1.04–1.9)^c^	5	3.0 (1.5–8.5)	4.7 (1.3–15.6)^c^	1.1 (0.4–2.1)
sevelamer	27	52.0 (9.0–68.0)	44.0 (29.9–63.4)^c^	1.1 (0.8–1.6)	5	39.0 (3.5–52.5)	34.4 (8.2–140.5)^c^	1.0 (0.4–2.3)	13	55.0 (10.0–74.0)	51.9 (32.5–80.7)^c^	1.5 (0.8–2.4)	10	51.0 (9.8–68.3)	48.9 (28.1–83.8)^c^	1.5 (0.7–2.5)
lanthanum carbonate	19	21.0 (6.0–78.0)	39.4 (23.6–63.8)^c^	1.0 (0.7–1.5)	1	4.0 (4.0–4.0)	-	-	7	72.0 (14.0–88.0)	60.1 (30.8–113.1)^c^	1.6 (0.7–3.1)	11	13.0 (6.0–47.0)	33.2 (16.1–65.2)^c^	1.0 (0.6–1.6)
colestilan	0	-	-	-	0	-	-	-	0	-	-	-	0	-	-	-
ferric citrate	3	54.0 (15.0–61.0)	44.1 (36.8–53.4)^c^	14.5 (3.2–39.2)^c^	0	-	-	-	2	57.5 (54.0–61.0)	-	-	1	15.0 (15.0–15.0)	-	-
Drugs for urinary frequency and incontinence (ATC^b^ code: G04BD)	41	13.0 (7.5–32.0)	23.4 (17.0–31.9)^c^	1.1 (0.8–1.4)	15	12.0 (4.0–31.0)	22.8 (12.2–40.8)^c^	1.0 (0.7–1.5)	2	9.0 (9.0–9.0)	-	-	24	14.5 (9.0–39.0)	24.7 (16.3–36.8)^c^	1.1 (0.8–1.5)
flavoxate	0	-	-	-	0	-	-	-	0	-	-	-	0	-	-	-
oxybutynin	2	52.0 (47.0–57.0)	-	-	0	-	-	-	0	-	-	-	2	52.0 (47.0–57.0)	-	-
propiverine	14	13.0 (6.0–35.0)	23.5 (13.0–41.0)^c^	1.1 (0.7–1.6)	6	9.5 (1.5–23.5)	17.4 (5.8–49.9)^c^	1.1 (0.5–1.9)	0	-	-	-	8	15.5 (9.0–39.0)	28.4 (12.8–59.9)^c^	1.2 (0.6–1.9)
tolterodine	5	13.0 (6.5–29.0)	23.9 (11.7–47.7)^c^	1.9 (0.8–3.5)	3	13.0 (0.0–42.0)	31.2 (8.6–121.2)^c^	2.0 (0.4–5.5)	0	-	-	-	2	14.5 (13.0–16.0)	-	-
solifenacin	10	10.0 (5.0–25.5)	20.5 (8.3–47.8)^c^	0.9 (0.5–1.4)	3	14.0 (6.0–88.0)	45.5 (3.5–671.6)^c^	1.0 (0.2–2.8)	0	-	-	-	7	9.0 (2.0–17.0)	15.5 (5.5–41.2)^c^	1.0 (0.5–1.7)
fesoterodine	5	9.0 (5.5–31.0)	16.6 (5.2–50.2)^c^	1.2 (0.5–2.6)	3	9.0 (4.0–31.0)	17.6 (2.8–123.9)^c^	1.4 (0.3–3.8)	0	-	-	-	2	19.0 (7.0–31.0)	-	-
mirabegron	5	13.0 (9.0–40.0)	20.3 (1.8–145.5)^c^	0.9 (0.3–1.8)	1	13.0 (13.0–13.0)	-	-	2	9.0 (9.0–9.0)	-	-	3	15.0 (9.0–65.0)	31.8 (2.7–421.4)^c^	1.1 (0.2–2.9)
Natural opium alkaloids (ATC^b^ code: N02AA)	67	12.0 (6.0–32.0)	21.1 (16.3–27.1)^c^	1.0 (0.9–1.2)	3	5.0 (0.0–18.0)	13.0 (3.2–57.3)^c^	1.9 (0.4–5.1)	13	13.0 (9.0–29.5)	15.0 (6.9–30.9)^c^	0.9 (0.5–1.4)	43	10.0 (4.0–27.0)	20.0 (14.0–28.1)^c^	1.0 (0.7–1.2)
morphine	11	13.0 (3.0–27.0)	20.9 (10.0–41.8)^c^	1.0 (0.6–1.5)	1	0.0 (0.0–0.0)	-	-	216.5 (13.0–20.0)	-	-	8	13.0 (3.3–31.5)	20.8 (6.8–59.5)^c^	0.8 (0.4–1.4)	
opium	0	-	-	-	0	-	-	-	0	-	-	-	0	-	-	-
oxycodone	56	9.5 (6.0–32.8)	21.2 (16.0–27.7)^c^	1.0 (0.8–1.8)	5	29.0 (11.5–48.0)	34.7 (20.4–58.1)^c^	2.7 (0.99–5.7)	11	9.0 (9.0–37.0)	15.6 (6.0–37.8)^c^	0.8 (0.5–1.4)	38	8.5 (4.0–19.0)	18.0 (12.2–26.2)^c^	0.9 (0.7–1.2)
Non-selective monoamine reuptake inhibitors (ATC^b^ code: N06AA)	10	19.0 (7.0–47.5)	31.6 (19.8–48.7)^c^	2.1 (1.02–3.6)^c^	0	-	-	-	0	-	-	-	10	19.0 (7.0–47.5)	31.6 (19.8–48.7)^c^	2.1 (1.02–3.6)^c^
imipramine	2	52.0 (52.0–52.0)	-	-	0	-	-	-	0	-	-	-	2	52.0 (52.0–52.0)	-	-
clomipramine	0	-	-	-	0	-	-	-	0	-	-	-	0	-	-	-
amitriptyline	6	13.0 (7.0–39.3)	30.5 (15.5–59.4)^c^	2.7 (0.8–6.3)	0	-	-	-	0	-	-	-	6	13.0 (7.0–39.3)	30.4 (15.5–59.4)^c^	2.7 (0.8–6.3)
nortriptyline	0	-	-	-	0	-	-	-	0	-	-	-	0	-	-	-
amoxapine	2	19.0 (19.0–19.0)	-	-	0	-	-	-	0	-	-	-	2	19.0 (19.0–19.0)	-	-
maprotiline	0	-	-	-	0	-			0	-	-	-	0	-	-	-
Oral bowel cleanser	145	0.0 (0.0–0.0)	1.6 (1.1–2.2)^c^	2.3 (1.4–3.4)^c^	64	0.0 (0.0–0.0)	1.7 (0.8–3.7)	3.5 (0.8–9.3)	50	0.0 (0.0–0.0)	1.3 (0.95–1.7)	3.2 (1.7–4.9)^c^	33	0.0 (0.0–0.0)	-	-
sodium potassium combination (including sodium potassium ascorbic acid combination agent)	145	0.0 (0.0–0.0)	1.6 (1.1–2.2)^c^	2.3 (1.4–3.4)^c^	64	0.0 (0.0–0.0)	1.7 (0.8–3.7)	3.5 (0.8–9.3)	51	0.0 (0.0–0.0)	1.3 (0.95–1.7)	3.2 (1.7–4.9)^c^	33	0.0 (0.0–0.0)	-	-
Other antineoplastic agents (ATC^b^ code: L01XX)	32	16.5 (8.3–46.3)	26.1 (18.2–36.8)^c^	1.1 (0.8–1.5)	4	10.0 (4.3–12.0)	8.4 (5.9–11.8)^c^	5.4 (1.6–13.4)^c^	25	23.0 (11.0–53.0)	31.1 (21.3–44.4)^c^	1.2 (0.8–1.6)	3	12.0 (7.0–17.0)	8.4 (3.9–18.7)^c^	3.5 (0.8–9.3)
asparaginase	2	9.5 (7.0–12.0)	-	-	0	-	-	-	0	-	-	-	2	9.5 (7.0–12.0)	-	-
hydroxycarbamide	0	-	-	-	0	-	-	-	0	-	-	-	0	-	-	-
estramustine	0	-	-	-	0	-	-	-	0	-	-	-	0	-	-	-
celecoxib	18	26.5 (12.0–52.8)	33.5 (22.4–48.8)^c^	1.4 (0.9–2.0)	2	12.0 (12.0–12.0)	-	-	16	29.5 (13.8–56.3)	37.0 (24.9–53.6)^c^	1.5 (0.9–2.2)	0	-	-	-
anagrelide	0	-	-	-	0	-	-	-	0	-	-	-	0	-	-	-
eribulin	3	14.0 (5.0–17.0)	11.2 (8.1–15.6)^c^	8.3 (1.8–22.5)^c^	0	-	-	-	2	9.5 (5.0–14.0)	-	-	1	17.0 (17.0–17.0)	-	-
aflibercept	9	14.0 (7.5–39.0)	24.0 (10.2–53.1)^c^	1.0 (0.6–1.6)	2	5.5 (3.0–8.0)	-	-	719.0 (9.0–55.0)	26.0 (8.9–71.6)^c^	1.0 (0.5–1.7)	0	-	-	-	
Other antipsychotics (ATC^b^ code: N05AX)	66	8.0 (2.0–15.0)	15.2 (11.2–20.5)^c^	0.9 (0.7–1.0)	2	8.0 (8.0–8.0)	-	-	0	-	-	-	39	8.0 (7.0–15.0)	17.5 (12.3–24.7)^c^	1.0 (0.8–1.2)
risperidone	21	8.0 (8.0–9.0)	9.6 (7.1–12.9)^c^	1.6 (1.1–2.1)^c^	1	8.0 (8.0–8.0)	-	-	0	-	-	-	22	8.0 (7.5–8.5)	9.3 (6.9–12.5)^c^	1.6 (1.1–2.1)^c^
mosapramine	0	-	-	-	0	-	-	-	0	-	-	-	0	-	-	-
zotepine	14	2.0 (2.0–2.0)	-	-	1	2.0 (2.0–2.0)	-	-	0	-	-	-	15	2.0 (2.0–2.0)	24.7 (10.1–59.8)^c^	1.9 (0.6–4.1)
aripiprazole	24	14.0 (7.0–52.0)	29.7 (18.6–46.1)^c^	1.0 (0.7–1.4)	2	18.5 (8.0–29.0)	-	-	0	-	-	-	22	14.0 (5.8–52.0)	30.1 (18.5–49.1)^c^	1.0 (0.7–1.4)
paliperidone	6	15.0 (6.3–17.3)	16.1 (11.0–23.2)^c^	3.1 (1.4–5.5)^c^	1	8.0 (8.0–8.0)	-	-	0	-	-	-	5	15.0 (8.0–19.5)	17.8 (12.9–24.3)^c^	4.2 (1.7–7.9)^c^
brexpiprazole	1	85.0 (85.0–85.0)	-	-	0	-	-	-	0	-	-	-	1	85.0 (85.0–85.0)	-	-
Phenothiazines with aliphatic side-chain (ATC^b^ code: N05AA)	45	8.0 (4.0–8.0)	10.7 (7.0–15.9)^c^	0.9 (0.7–1.1)	310.0 (8.0–10.0)	-	-	0	-	-	-	18	8.0 (8.0–34.3)	40.4 (24.3–65.5)^c^	2.1 (0.96–3.7)	
chlorpromazine	17	4.0 (4.0–10.0)	29.7 (11.9–70.6)^c^	1.3 (0.6–2.6)	3	10.0 (4.0–10.0)	-	-	0	-	-	-	15	4.0 (4.0–4.0)	45.8 (30.8–67.8)^c^	4.5 (1.4–9.8)^c^
levomepromazine	28	8.0 (8.0–8.0)	34.6 (14.2–82.0)^c^	1.6 (0.6–3.0)	2	8.0 (8.0–8.0)	-	-	0	-	-	-	27	8.0 (8.0–8.0)	37.5 (10.9–127.7)^c^	1.5 (0.5–3.2)
Poly (ADP-ribose) polymerase (PARP) inhibitors (ATC^b^ code: L01XK)	4	16.5 (11.0–38.5)	17.1(4.0–72.3)^c^	1.2 (0.4–2.6)	2	32.0 (19.0–45.0)	-	-	1	10.0 (10.0–10.0)	-	-	1	14.0 (14.0–14.0)	-	-
olaparib	4	16.5 (11.0–38.5)	17.1(4.0–72.3)^c^	1.2 (0.4–2.6)	2	32.0 (19.0–45.0)	-	-	1	10.0 (10.0–10.0)	-	-	1	14.0 (14.0–14.0)	-	-
Proteasome inhibitors (ATC^b^ code: L01XG)	53	13.0 (6.0–30.5)	24.0 (18.1–31.4)^c^	1.1 (0.9–1.4)	3	17.0 (12.0–49.0)	22.3 (2.5–225.8)^c^	1.2 (0.3–3.2)	6	48.5 (9.0–64.3)	45.7 (22.8–90.2)^c^	1.8 (0.7–3.7)	44	13.0 (4.0–26.8)	2.9 (2.6–3.3)^c^	1.0 (0.8–1.3)
bortezomib	47	13.0 (6.0–31.0)	22.9 (16.7–30.9)^c^	1.0 (0.8–1.3)	2	30.5 (12.0–49.0)	-	-	5	52.0 (8.0–65.5)	45.9 (17.0–121.1)^c^	1.5 (0.5–3.4)	40	13.0 (4.0–23.0)	20.2 (14.3–28.1)^c^	1.1 (0.8–1.3)
Ixazomib	6	26.5 (13.0–33.8)	31.2 (21.9–43.8)^c^	3.3 (1.5–5.8)^c^	1	17.0 (17.0–17.0)	-	-	1	45.0 (45.0–45.0)	-	-	4	26.5 (7.3–29.3)	27.5 (24.7–30.5)^c^	16.4 (5.4–35.0)^c^
Topoisomerase 1 (TOP1) inhibitors (ATC^b^ code: L01CE)	157	19.0 (8.0–39.5)	27.8 (24.2–32.0)^c^	1.2 (1.1–1.4)^c^	2616.5 (7.8–53.0)	24.6 (15.6–37.8)^c^	1.0 (0.7–1.3)	84 21.0 (8.3–40.8)	28.4 (23.5–34.1)^c^	1.2 (1.04–1.5)^c^	4616.5 (7.8–36.0)	26.1 (19.7–34.2)^c^	1.1 (0.9–1.4)			
irinotecan	157	19.0 (8.0–39.5)	27.8 (24.2–32.0)^c^	1.2 (1.1–1.4)^c^	26	16.5 (7.8–53.0)	24.6 (15.6–37.8)^c^	1.0 (0.7–1.3)	84	21.0 (8.3–40.8)	28.4 (23.5–34.1)^c^	1.2 (1.04–1.5)^c^	46	16.5 (7.8–36.0)	26.1 (19.7–34.2)^c^	1.1 (0.9–1.4)
Vinca alkaloids and analogues (ATC^b^ code: L01CA)	72	8.0 (4.0–20.0)	16.4 (12.6–21.2)^c^	1.0 (0.8–1.2)	6	5.0 (2.8–46.5)	16.4 (2.8–86.6)^c^	0.7 (0.3–1.3)	25	7.0 (3.0–21.0)	16.4 (9.8–26.9)^c^	0.9 (0.6–1.2)	37	11.0 (5.5–17.5)	16.3 (11.6–22.5)^c^	1.1 (0.8–1.3)
vinblastine	2	12.5 (3.0–22.0)	-	-	0	-	-	-	0	-	-	-	2	12.5 (3.0–22.0)	-	-
vincristine	51	9.0 (4.0–20.0)	17.6 (12.8–23.7)^c^	1.0 (0.8–1.2)	4	23.5 (4.5–57.5)	32.3 (4.7–218.7)^c^	1.0 (0.3–2.3)	20	7.0 (3.0–20.0)	16.5 (8.9–29.7)^c^	0.9 (0.6–1.2)	25	11.0 (6.5–17.5)	15.9 (10.6–23.4)^c^	1.1 (0.8–1.5)
vindesine	1	8.0 (8.0–8.0)	-	-	0	-	-	-	0	-	-	-	1	8.0 (8.0–8.0)	-	-
vinorelbine	18	6.0 (3.8–14.3)	14.0 (7.8–24.3)^c^	0.9 (0.6–1.3)	2	2.5 (2.0–3.0)	-	-	5	5.0 (4.5–31.5)	11.5 (1.5–78.8)^c^	0.7 (0.3–1.4)	11	7.0 (5.0–12.0)	16.1 (7.5–33.3)^c^	1.0 (0.6–1.4)

a CI: Confidence interval

b ATC: the Anatomical Therapeutic Chemical (ATC) classification system described by the World Health Organization Collaborating Centre for Drug Statistics Methodology (www.whocc.no/atc_ddd_index)

c Lower limit of 95% CI > 1 Number of cases <2

**FIGURE 3:**
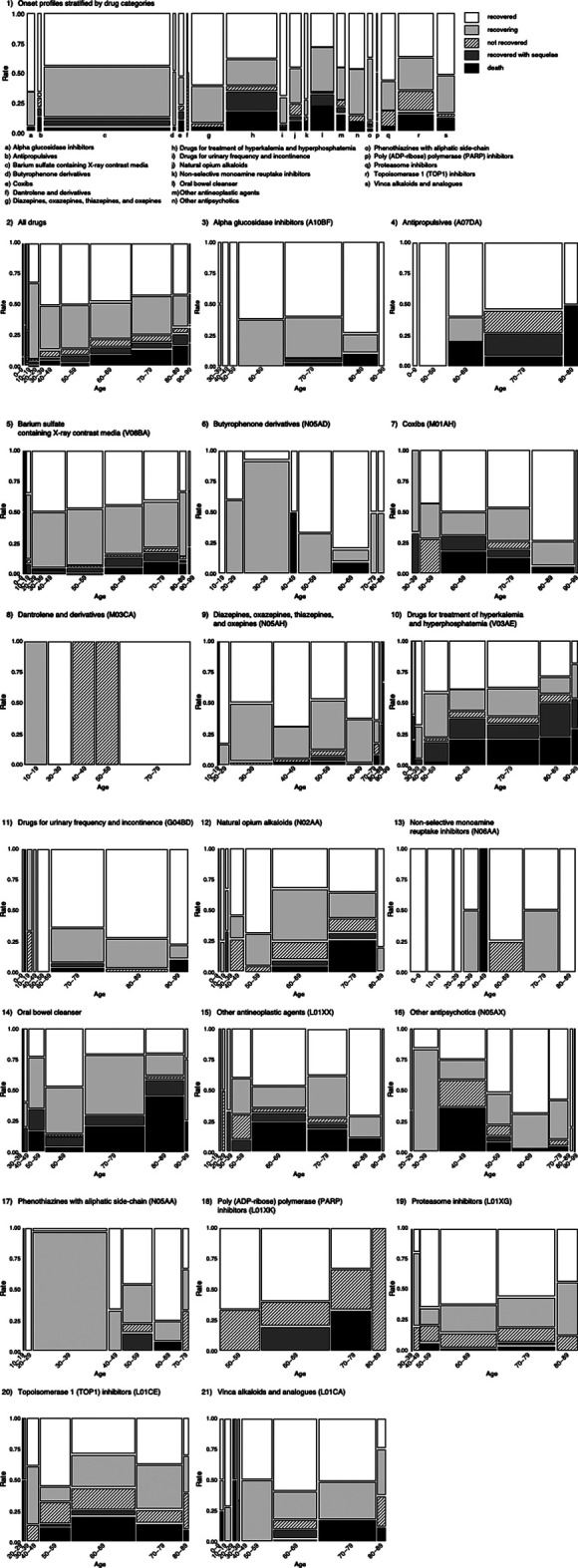
Mosaic plot of drug-induced gastrointestinal obstruction and perforation.

We used a mosaic plot to summarize the outcome profiles of DIGs encompassed by the 19 categories (Figure 3). It is clear from the plot that antipropulsives, coxibs, drugs for treatment of hyperkalemia and hyperphosphatemia, oral bowel cleansers, other antineoplastic agents, and TOP1 inhibitors were associated with death in more than 10% of the cases in each category. The reporting ratios of death outcomes increased due to aging.

The results of drug signal detection, time-to-onset analysis, and evaluation of outcome using the PTs for *ileus*, *obstruction*, and *perforation* are summarized in Table 1, 2, Supplementary Figures S2–S4, and Supplementary Figures S5–S7, respectively.

## Discussion

Here we report that AE signals for DIGs were detected for several drugs in the JADER database. The risk of DIGs due to α-glucosidase inhibitors, antipropulsives, antipsychotics, barium sulfate containing X-ray contrast media, dantrolene and derivatives, drugs for treatment of hyperkalemia and hyperphosphatemia, drugs for urinary frequency and incontinence, natural opium alkaloids, non-selective monoamine reuptake inhibitors, oral bowel cleansers, and other antineoplastic agents have been described in several reports (Harel et al., 2013; Every-Palmer et al., 2017; EZEM Canada Inc., 2017; De Berardis et al., 2018), and are in agreement with our results from the present study.

To understand the characteristics of DIGs, the time-to-onset profile of DIGs is important. The time between treatment with an α-glucosidase inhibitor and the occurrence of ileus is reported to range from several days to three months (Hayaishi et al., 1996; Oba et al., 2001). The median onset time for α-glucosidase inhibition was 16.5 days in our study. α-Glucosidase inhibitors may induce bowel obstruction, which is a risk factor for ileus in patients undergoing abdominal surgery, and these drugs should be administered carefully (Ministry of Health, Labour and Welfare, 2008; Vilz et al., 2017).

Constipation caused by reduced gastrointestinal motility is commonly associated with many antipsychotics (Ministry of Health, Labour and Welfare, 2008; Every-Palmer and Ellis, 2017; Every-Palmer et al., 2017). Gastrointestinal motility is reduced by the anticholinergic effect resulting from blockade of muscarinic receptors by antipsychotics (Nielsen and Meyer, 2012). The anticholinergic potency differs among antipsychotics (Nielsen and Meyer, 2012). Clozapine has a high affinity for muscarinic receptors and is associated with a significantly higher incidence of constipation compared to other antipsychotics (Every-Palmer et al., 2017). It was reported that the onset of ileus occurred, on an average, more than 3 years after the first prescription of drugs such as clozapine, first-generation antipsychotics, tricyclic antidepressants, and anticholinergics (Nielsen and Meyer, 2012). Other researchers have reported that gastrointestinal hypomotility can occur at any stage of treatment with clozapine (Every-Palmer and Ellis, 2017). In our study, the median onset of ileus following treatment with “diazepines, oxazepines, thiazepines, and oxepines” and “clozapine” was 8 and 20 days, respectively.

Retention of barium sulfate in the digestive tract rarely causes gastrointestinal perforation, intestinal obstruction, colon ulcer, colitis, diverticulitis, and barium appendicitis. More severe outcomes may be observed in the elderly (EZEM Canada Inc, 2017), which is consistent with our results.

Because drugs classed as “muscle relaxants, directly acting agents,” such as dantrolene, may worsen symptoms due to the muscle relaxation effect, they should be administered carefully to patients with ileus (Eagle Pharmaceuticals, 2017).

Drugs for treatment of hyperkalemia and hyperphosphatemia, such as polystyrene sulfonic acid preparation, may stagnate in the intestinal tract and this is followed by solidification of the intestinal content, causing ileus-like symptoms (Ministry of Health, Labour and Welfare, 2008). Similar drugs, such as sodium polystyrene sulfonate and orally administered sorbitol suspension of polystyrene sulfonate, have also been reported to cause perforation of the small intestine, intestinal mucosal necrosis, colon ulcer, colon necrosis, and other symptoms (Harel et al., 2013). There is an association between polystyrene sulfonate use and hospitalization or emergency department visit for an adverse gastrointestinal event within 30 days (Noel et al., 2019). Time to event after the administration of sodium polystyrene sulfonate is 12 days (Noel et al., 2019). It has also been reported that gastrointestinal symptoms manifest shortly after the administration of sodium polystyrene (median 2 days) (Harel et al., 2013). Sodium polystyrene sulfate use may be associated with fatal gastrointestinal injury (Ministry of Health, Labour and Welfare, 2008; Harel et al., 2013; Noel et al., 2019), which is consistent with our results. The median onset date of DIGs associated with sevelamer hydrochloride (52 days) was different from that of sodium polystyrene sulfonate. For sevelamer hydrochloride, DIGs, if detected, need to be monitored for several months.

Anti-cholinergic drugs, such as “drugs for urinary frequency and incontinence,” suppress contractile movement of intestinal smooth muscle through their anticholinergic action, and the intestinal content becomes stagnant due to a decrease in intestinal tone, resulting in paralytic ileus and flaccid constipation (Ministry of Health, Labor and Welfare, 2008). The median onset date for this condition was found to be 13 days.

The action of opium alkaloids on the *μ* receptor results in the suppression of gastrointestinal motility. Thus, the passage of gastric content is delayed, leading to the solidification of the stool. Furthermore, constipation occurs due to tension in the anal sphincter and suppression of the central defecation reflex (Ministry of Health, Labour and Welfare, 2008). The median onset date for this was found to be 12 days.

Oral bowel cleansers may cause intestinal perforation due to elevated intestinal pressure. This may lead to more severe outcomes in the elderly (Ajinomoto Pharma Co, Ltd, 2003). Oral bowel cleansers were reported to be associated with the development of symptoms of ileus and intestinal perforation several hours after administration (Ajinomoto Pharma Co, Ltd, 2003). Our results highlight the effect of age and are consistent with previously reported findings.

Although it is not clear how anticancer drugs cause paralytic ileus, peripheral (autonomic) neuropathy, which is frequently caused by microtubule inhibitors (vinca alkaloids) (Hancock and Naysmith, 1975) and bortezomib (Sonneveld and Jongen, 2010), may be involved in severe constipation with ileus. Patients with cancer are affected by various factors, such as lack of exercise, old age, depression, low residue diet, muscle weakness, pain, surgery, gastrointestinal imaging with barium sulfate, and medication (diuretic dehydration, anticholinergics, antidepressants, analgesics, etc.) and they tend to develop constipation and paralytic ileus (Ministry of Health, Labor and Welfare, 2008). AEs of anticancer drugs are also affected by the type of cancer and the treatment protocol. It is important to evaluate each risk factor statistically. However, SRS lacks detailed information on patient background. Further epidemiological studies using large sample sizes and well-controlled prospective clinical trials in which confounding factors are controlled will be required for obtaining clarity on the association of anticancer drug use and paralytic ileus.

It is important that general practitioners and gastroenterologists know the actual timing and outcome profiles of DIGs, based on real-world clinical data. In this way, early intervention in AEs is possible and the risk of overlooking DIGs is reduced. In this study, we did not directly verify pharmacological findings. Attempts have been made in the area of drug repositioning to search for genes related to AEs inspired by simple ROR values. Application to such trials will be undertaken in a future study.

Generally, there are few stakeholders who actively promote prospective clinical research to evaluate the risk of AEs. Therefore, retrospective studies of AE risk using electronic medical records are considered valuable; however, they are more susceptible to bias than prospective intervention studies. Therefore, the AE profile survey using SRS has certain value. The JADER database is the largest primary data tool available to regulatory authorities for pharmacovigilance of AEs post-marketing. It represents an opportunity to interrogate data that reflect the realities of clinical practice. Our results are consistent with those reported in the literature, and they are essential to strengthening and expanding our existing knowledge. Our study highlights the importance of comparing safety profiles using post-marketing real-world data.

Our analysis was restricted to reports in which drugs were recorded as “suspected drug.” The RORs might vary depending on the selection of PTs that were assigned causality by contributors. The data in the SRS database have been reported by healthcare “professionals.” With a narrow selection of PT-related DIGs, the identification of cases is highly likely to represent the condition of interest. More AE reports from Japan having high completeness were submitted by physicians and included a single AE term (Wakao et al., 2019). We believe that these results are worthy of evaluation and that these data suggest the association of certain drugs with DIGs.

AE reporting profiles may vary between countries owing to differences in population composition, treatment policies, and regulations of administrative authorities in each country. Wakao *et al.* (2019) clarified the characteristics of Japanese AE reports compared to other countries based on an analysis of VigiBase, which shares serious reports with the World Health Organization (WHO) Program for International Drug Monitoring (Wakao et al., 2019). They found that 10 top AEs, including interstitial lung disease, abnormal hepatic function, decreased platelet count, decreased neutrophil count, and drug eruption, had higher relative reporting rates in Japan compared to global reporting rates. They also identified 10 top AEs with lower relative reporting rates in Japan. In comparison with the global reporting in VigiBase, Japanese people might not be susceptible to DIGs according to pharmacovigilance data.

Because the JADER database is an SRS, several limitations of the present analysis should be noted. The RORs may vary significantly depending on the selection of PTs. The SRS databases are subject to over-reporting, under-reporting, and comorbidities.

The SRS lacks a control population or reference group of healthy individuals. Therefore, ROR cannot be used for true risk assessment and provides a rough indication as a starting point for exploratory analyses.

The number of spontaneously reported cases has increased following safety alerts by regulatory authorities, which is a phenomenon known as notoriety bias (Pariente et al., 2007; De Bruin et al., 2002; Motola et al., 2008). The warning by the PMDA or pharmaceutical company might result in increased ROR. Warnings against DIGs caused by oral bowel cleanser and sevelamer were issued by a pharmaceutical company in 2003 (Ajinomoto Pharma Co, Ltd, 2003; CHUGAI PHARMACEUTICAL CO, LTD, 2003). Although these alerts were issued before 2004, the notoriety bias does not seem to affect our results, according to the JADER dataset used in this analysis. Furthermore, an epidemiological phenomenon wherein an increase in the number of AE reports coincides with the introduction of a drug by the regulatory authority is known as the Weber effect (Wallenstein and Fife, 2001; Hartnell and Wilson, 2004; McAdams et al., 2008). However, the Weber effect is not always observed (McAdams et al., 2008). Information on the reporting year related to the safety warning may have inflated the ROR, which is the index of disproportionality analysis. There are several reports on attempts to adjust the ROR by incorporating the item of the reporting year into the model formula of a multiple logistic analysis (Van Puijenbroek et al., 1999; Suzuki et al., 2015; Takeyama et al., 2017). Takeyama *et al.* (2017) evaluated the effectiveness of the year of regulatory actions using multivariate logistic regression analysis (Takeyama et al., 2017). However, we did not evaluate the subsets as before/after the PMDA regulation in this study.

The rate of severe outcome was low with barium sulfate, which has the highest ROR. On the other hand, a risk of death outcome greater than 10% was found for antipropulsives, coxibs, drugs for treatment of hyperkalemia and hyperphosphatemia, oral bowel cleanser, other antineoplastic agents, and TOP1 inhibitors. Antipropulsives, other antineoplastic agents, TOP1 inhibitors, and coxibs are linked to neoplasia and their therapies. The patient’s disease background and severity might affect outcome results and ROR values. It is difficult to exclude severe underlying disease as a cause of severe outcomes. We suggest that patient background, including disease, should be assessed in a structured manner and that analysis should include more complex interactions of the possible confounders. Propensity scores may be used to reduce bias by equating groups based on possible confounders (Wang et al., 2020; Akimoto et al., 2016). Alternately, there have been some recent approaches to deal with them in a high dimensional context (Schuemie et al., 2018; Hripcsak et al., 2016; Tian et al., 2018). To date, there is no widely accepted and established method for adjusting the covariates in studies using SRS dataset. Therefore, it is a subject for future study. Our results must be carefully interpreted considering the limitations of this study.

To the best of our knowledge, this is the first study to evaluate DIGs using the Japanese Adverse Drug Event Report database. Based on RORs, we demonstrated a potential risk for DIGs associated with several drugs, including α-glucosidase inhibitors, antipsychotics such as clozapine, barium sulfate, sevelamar, and oral bowel cleansers. Many drugs associated with DIGs have serious consequences. Depending on the drug, the time of onset of the DIG extends from days to several months. It is, therefore, necessary to perform detailed monitoring for each drug.

## Data Availability

The raw data supporting the conclusions of this article will be made available by the authors, without undue reservation.
