# Supplementary material for: Analysis of Drug-Induced Gastrointestinal Obstruction and Perforation Using the Japanese Adverse Drug Event Report Database
Source: Front Pharmacol. 2021 Jul 26;12:692292. doi: 10.3389/fphar.2021.692292 (PMC8350341; doi:10.3389/fphar.2021.692292)
Supplement: Supplementary file 2 [file Presentation1.PPTX]

## Slide 1
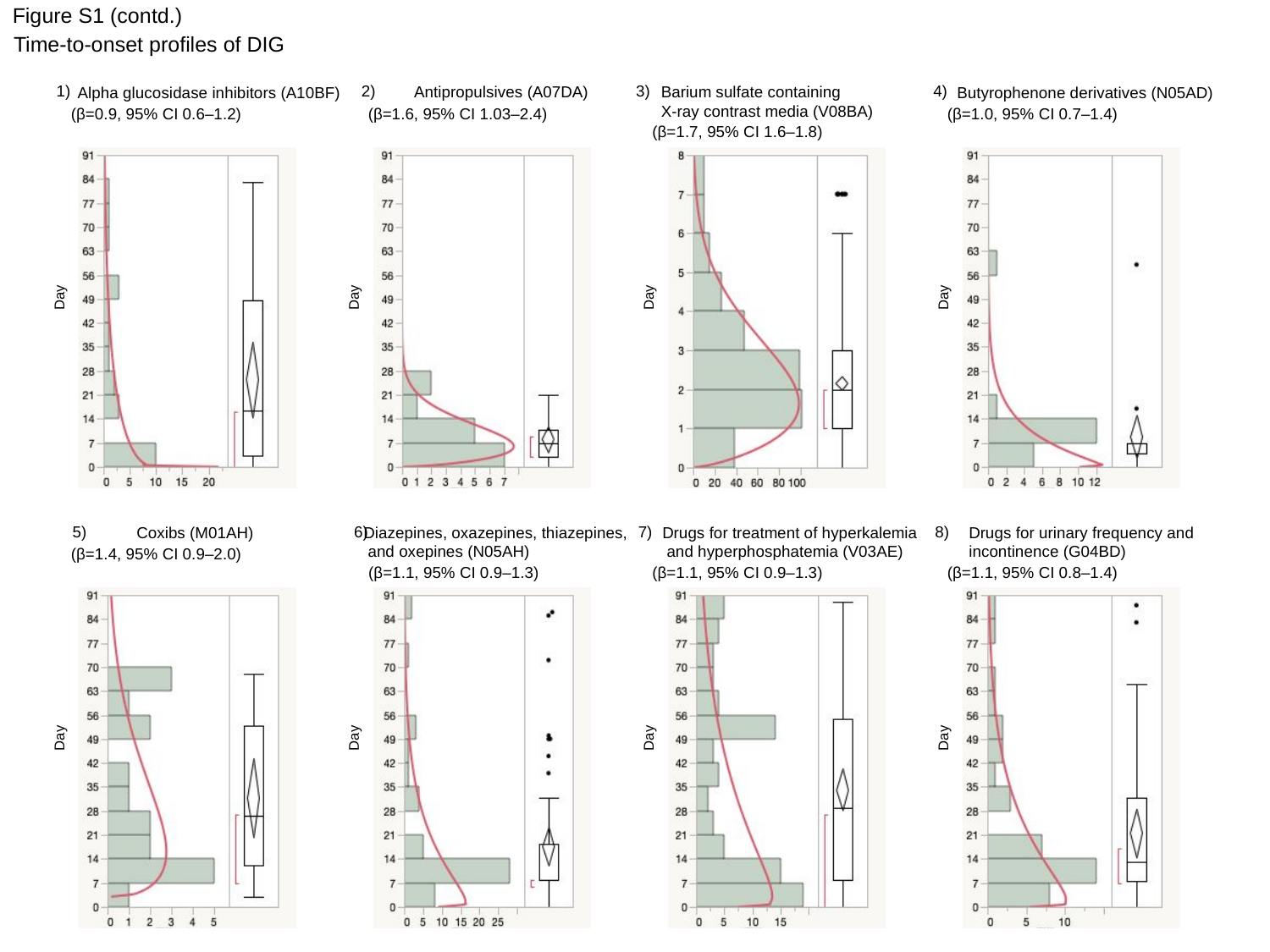

Figure S1 (contd.)
Time-to-onset profiles of DIG
1)
2)
3)
4)
Antipropulsives (A07DA)
Alpha glucosidase inhibitors (A10BF)
Barium sulfate containing
X-ray contrast media (V08BA)
Butyrophenone derivatives (N05AD)
(β=0.9, 95% CI 0.6–1.2)
(β=1.6, 95% CI 1.03–2.4)
(β=1.0, 95% CI 0.7–1.4)
(β=1.7, 95% CI 1.6–1.8)
Day
Day
Day
Day
5)
6)
7)
8)
Coxibs (M01AH)
Diazepines, oxazepines, thiazepines,
 and oxepines (N05AH)
Drugs for treatment of hyperkalemia
 and hyperphosphatemia (V03AE)
Drugs for urinary frequency and
incontinence (G04BD)
(β=1.4, 95% CI 0.9–2.0)
(β=1.1, 95% CI 0.9–1.3)
(β=1.1, 95% CI 0.9–1.3)
(β=1.1, 95% CI 0.8–1.4)
Day
Day
Day
Day

## Slide 2
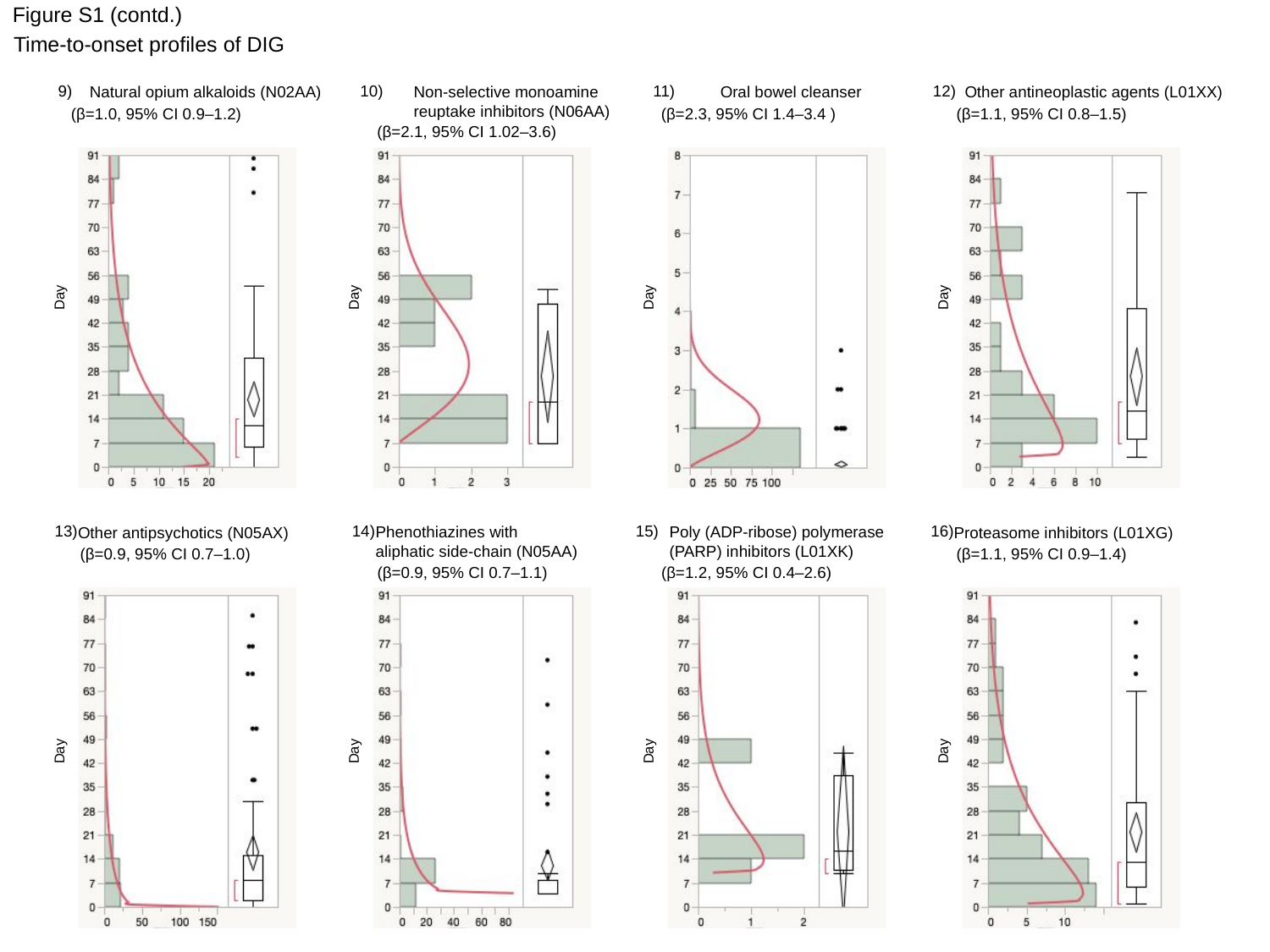

Figure S1 (contd.)
Time-to-onset profiles of DIG
9)
Natural opium alkaloids (N02AA)
10)
Non-selective monoamine
reuptake inhibitors (N06AA)
11)
Oral bowel cleanser
12)
Other antineoplastic agents (L01XX)
(β=1.0, 95% CI 0.9–1.2)
(β=2.3, 95% CI 1.4–3.4 )
(β=1.1, 95% CI 0.8–1.5)
(β=2.1, 95% CI 1.02–3.6)
Day
Day
Day
Day
13)
Other antipsychotics (N05AX)
14)
15)
16)
Phenothiazines with
aliphatic side-chain (N05AA)
Poly (ADP-ribose) polymerase
(PARP) inhibitors (L01XK)
Proteasome inhibitors (L01XG)
(β=0.9, 95% CI 0.7–1.0)
(β=1.1, 95% CI 0.9–1.4)
(β=0.9, 95% CI 0.7–1.1)
(β=1.2, 95% CI 0.4–2.6)
Day
Day
Day
Day

## Slide 3
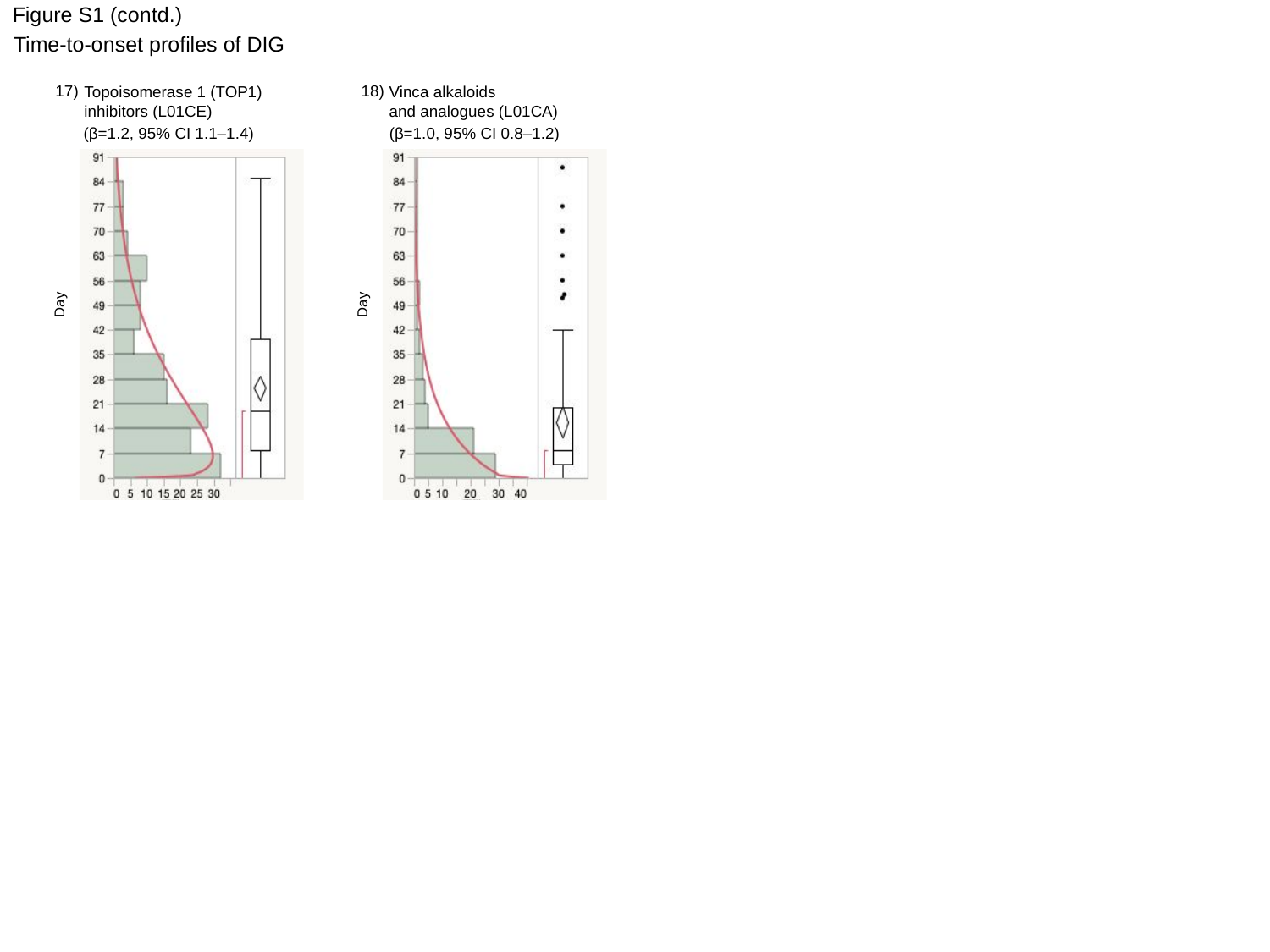

Figure S1 (contd.)
Time-to-onset profiles of DIG
17)
18)
Topoisomerase 1 (TOP1)
inhibitors (L01CE)
Vinca alkaloids
and analogues (L01CA)
(β=1.2, 95% CI 1.1–1.4)
(β=1.0, 95% CI 0.8–1.2)
Day
Day
