# Supplementary material for: Analysis of Drug-Induced Gastrointestinal Obstruction and Perforation Using the Japanese Adverse Drug Event Report Database
Source: Front Pharmacol. 2021 Jul 26;12:692292. doi: 10.3389/fphar.2021.692292 (PMC8350341; doi:10.3389/fphar.2021.692292)
Supplement: Supplementary file 3 [file Presentation4.PPTX]

## Slide 1
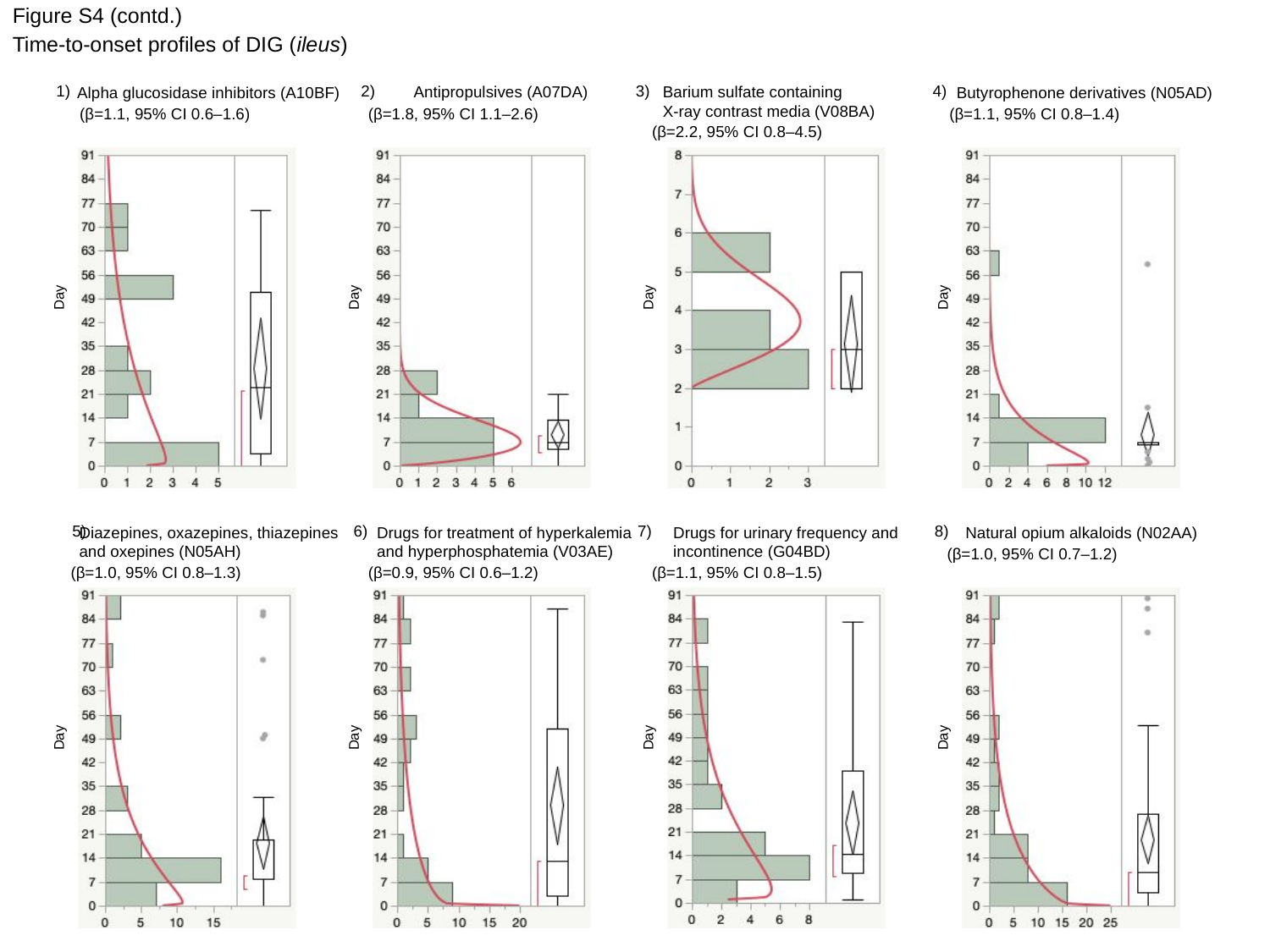

Figure S4 (contd.)
Time-to-onset profiles of DIG (ileus)
1)
2)
3)
4)
Antipropulsives (A07DA)
Alpha glucosidase inhibitors (A10BF)
Barium sulfate containing
X-ray contrast media (V08BA)
Butyrophenone derivatives (N05AD)
(β=1.1, 95% CI 0.6–1.6)
(β=1.8, 95% CI 1.1–2.6)
(β=1.1, 95% CI 0.8–1.4)
(β=2.2, 95% CI 0.8–4.5)
Day
Day
Day
Day
5)
6)
7)
8)
Diazepines, oxazepines, thiazepines
and oxepines (N05AH)
Drugs for treatment of hyperkalemia
and hyperphosphatemia (V03AE)
Drugs for urinary frequency and
incontinence (G04BD)
Natural opium alkaloids (N02AA)
(β=1.0, 95% CI 0.7–1.2)
(β=1.0, 95% CI 0.8–1.3)
(β=0.9, 95% CI 0.6–1.2)
(β=1.1, 95% CI 0.8–1.5)
Day
Day
Day
Day

## Slide 2
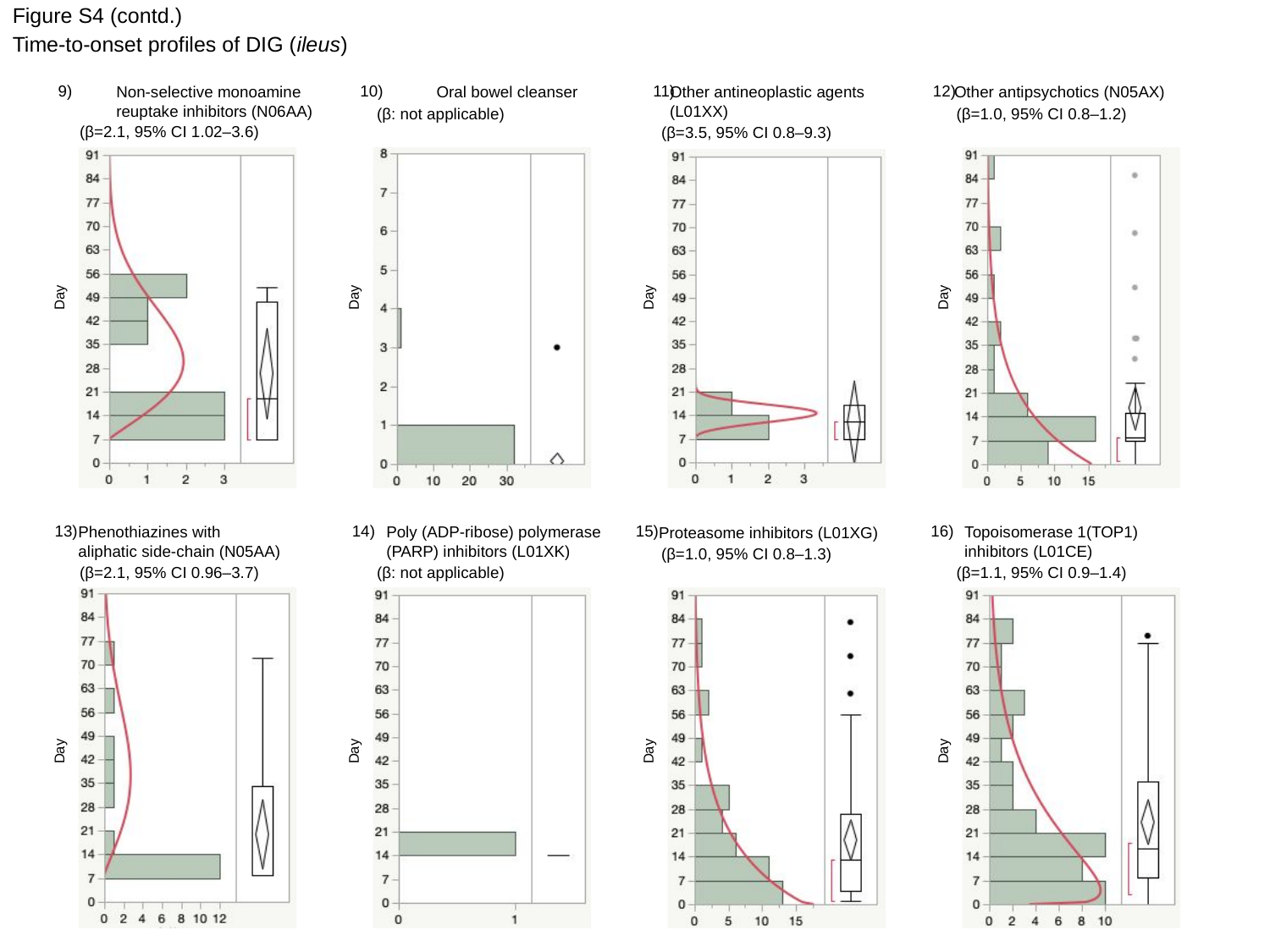

Figure S4 (contd.)
Time-to-onset profiles of DIG (ileus)
9)
10)
11)
12)
Non-selective monoamine
reuptake inhibitors (N06AA)
Oral bowel cleanser
Other antineoplastic agents
(L01XX)
Other antipsychotics (N05AX)
(β=1.0, 95% CI 0.8–1.2)
(β=2.1, 95% CI 1.02–3.6)
(β=3.5, 95% CI 0.8–9.3)
Day
Day
Day
Day
13)
14)
15)
16)
Phenothiazines with
aliphatic side-chain (N05AA)
Poly (ADP-ribose) polymerase
(PARP) inhibitors (L01XK)
Proteasome inhibitors (L01XG)
Topoisomerase 1(TOP1)
inhibitors (L01CE)
(β=1.0, 95% CI 0.8–1.3)
(β=2.1, 95% CI 0.96–3.7)
(β=1.1, 95% CI 0.9–1.4)
Day
Day
Day
Day
(β: not applicable)
(β: not applicable)

## Slide 3
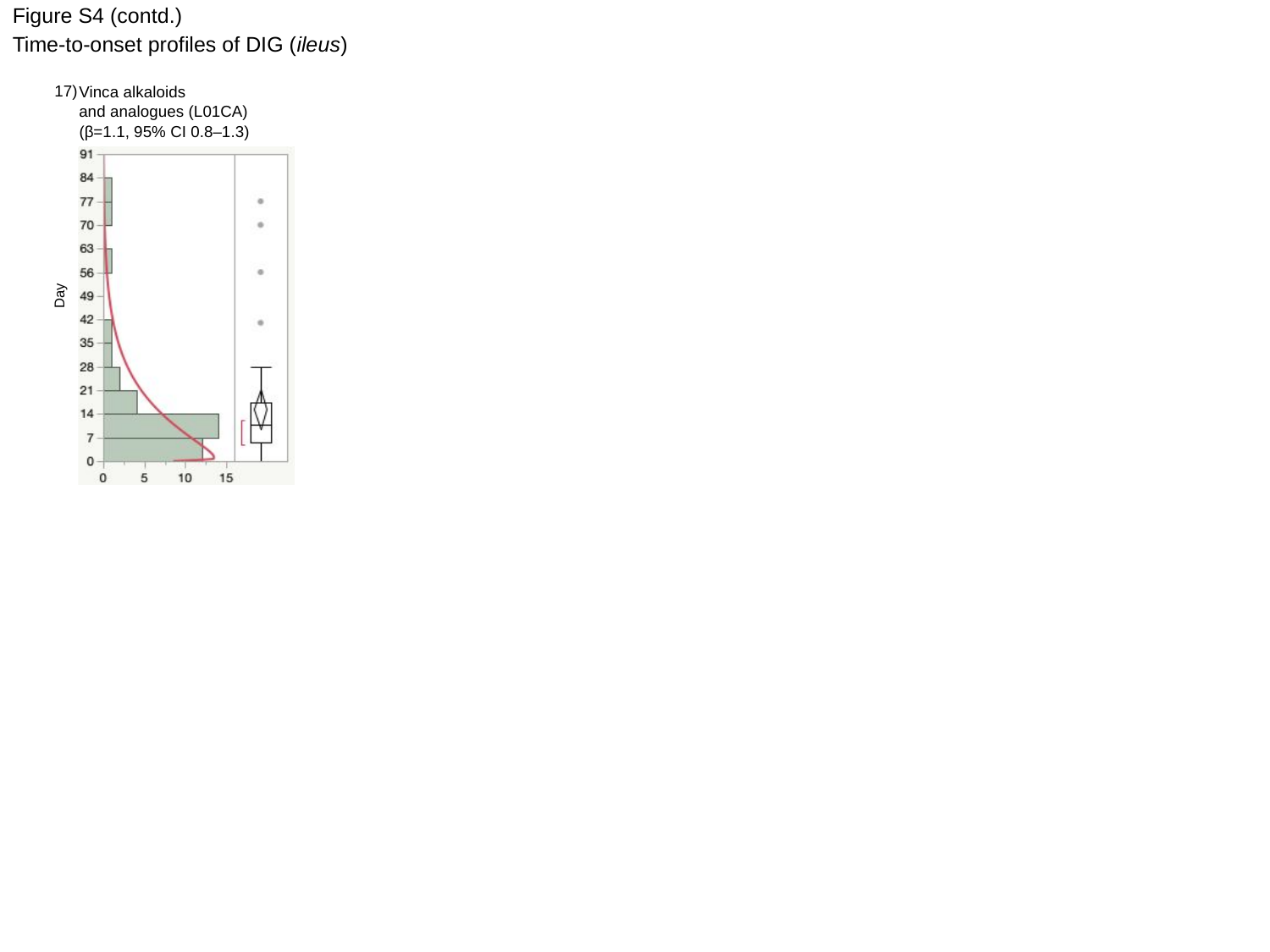

Figure S4 (contd.)
Time-to-onset profiles of DIG (ileus)
17)
Vinca alkaloids
and analogues (L01CA)
(β=1.1, 95% CI 0.8–1.3)
Day
