# Supplementary material for: Analysis of Drug-Induced Gastrointestinal Obstruction and Perforation Using the Japanese Adverse Drug Event Report Database
Source: Front Pharmacol. 2021 Jul 26;12:692292. doi: 10.3389/fphar.2021.692292 (PMC8350341; doi:10.3389/fphar.2021.692292)
Supplement: Supplementary file 4 [file Presentation6.PPTX]

## Slide 1
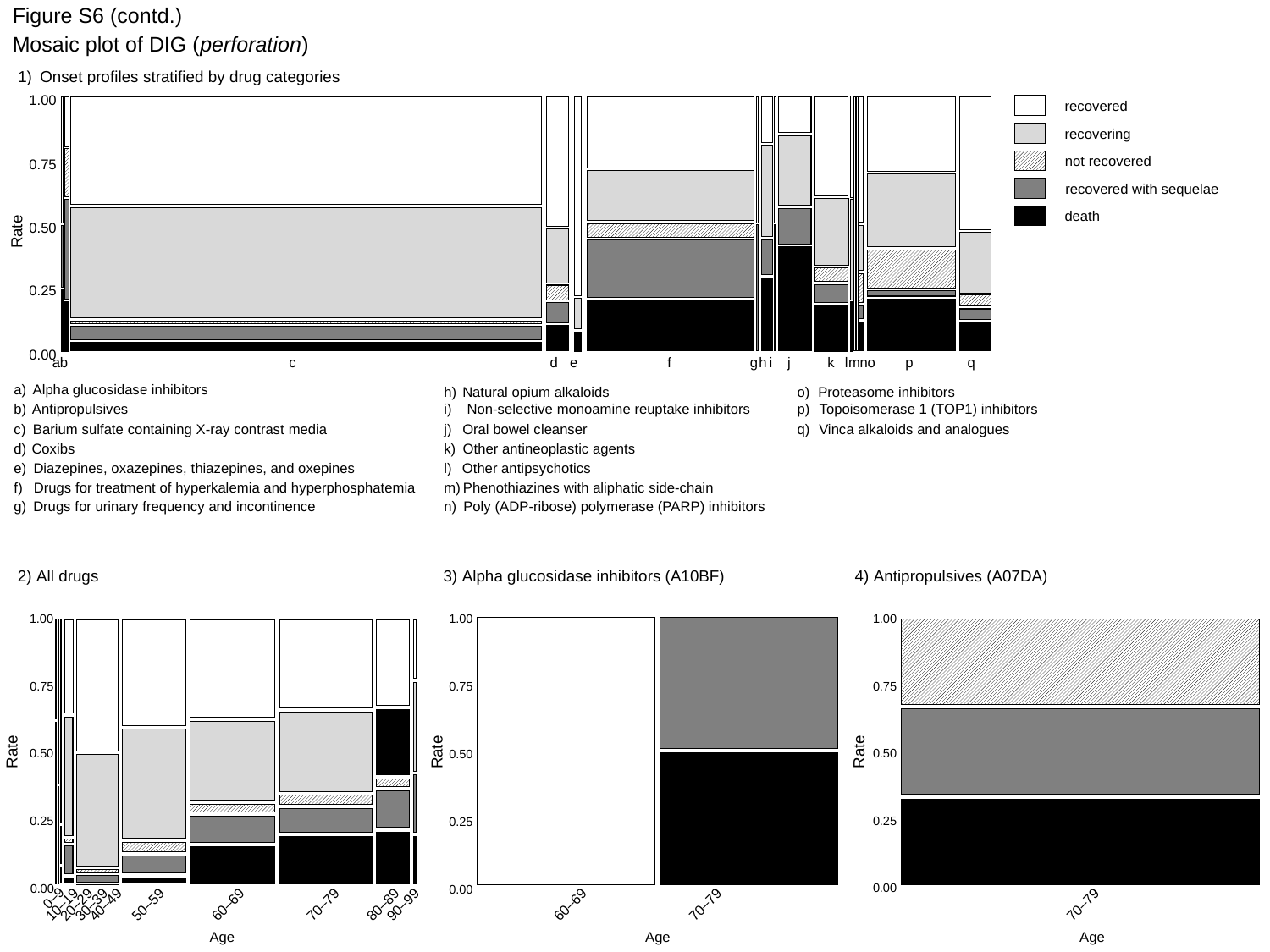

Figure S6 (contd.)
Mosaic plot of DIG (perforation)
1)
Onset profiles stratified by drug categories
1.00
0.75
0.50
0.25
0.00
Rate
recovered
recovering
not recovered
recovered with sequelae
death
a
b
c
d
e
f
g
h
i
j
k
l
m
n
o
p
q
a)
Alpha glucosidase inhibitors
b)
Antipropulsives
c)
Barium sulfate containing X-ray contrast media
d)
Coxibs
e)
Diazepines, oxazepines, thiazepines, and oxepines
f)
Drugs for treatment of hyperkalemia and hyperphosphatemia
g)
Drugs for urinary frequency and incontinence
h)
Natural opium alkaloids
i)
Non-selective monoamine reuptake inhibitors
j)
Oral bowel cleanser
k)
Other antineoplastic agents
l)
Other antipsychotics
m)
Phenothiazines with aliphatic side-chain
n)
Poly (ADP-ribose) polymerase (PARP) inhibitors
o)
Proteasome inhibitors
p)
Topoisomerase 1 (TOP1) inhibitors
q)
Vinca alkaloids and analogues
2)
All drugs
3)
Alpha glucosidase inhibitors (A10BF)
4)
Antipropulsives (A07DA)
1.00
0.75
0.50
0.25
0.00
Rate
1.00
0.75
0.50
0.25
0.00
Rate
1.00
0.75
0.50
0.25
0.00
Rate
0–9
10–19
20–29
30–39
40–49
50–59
60–69
70–79
80–89
90–99
60–69
70–79
70–79
Age
Age
Age

## Slide 2
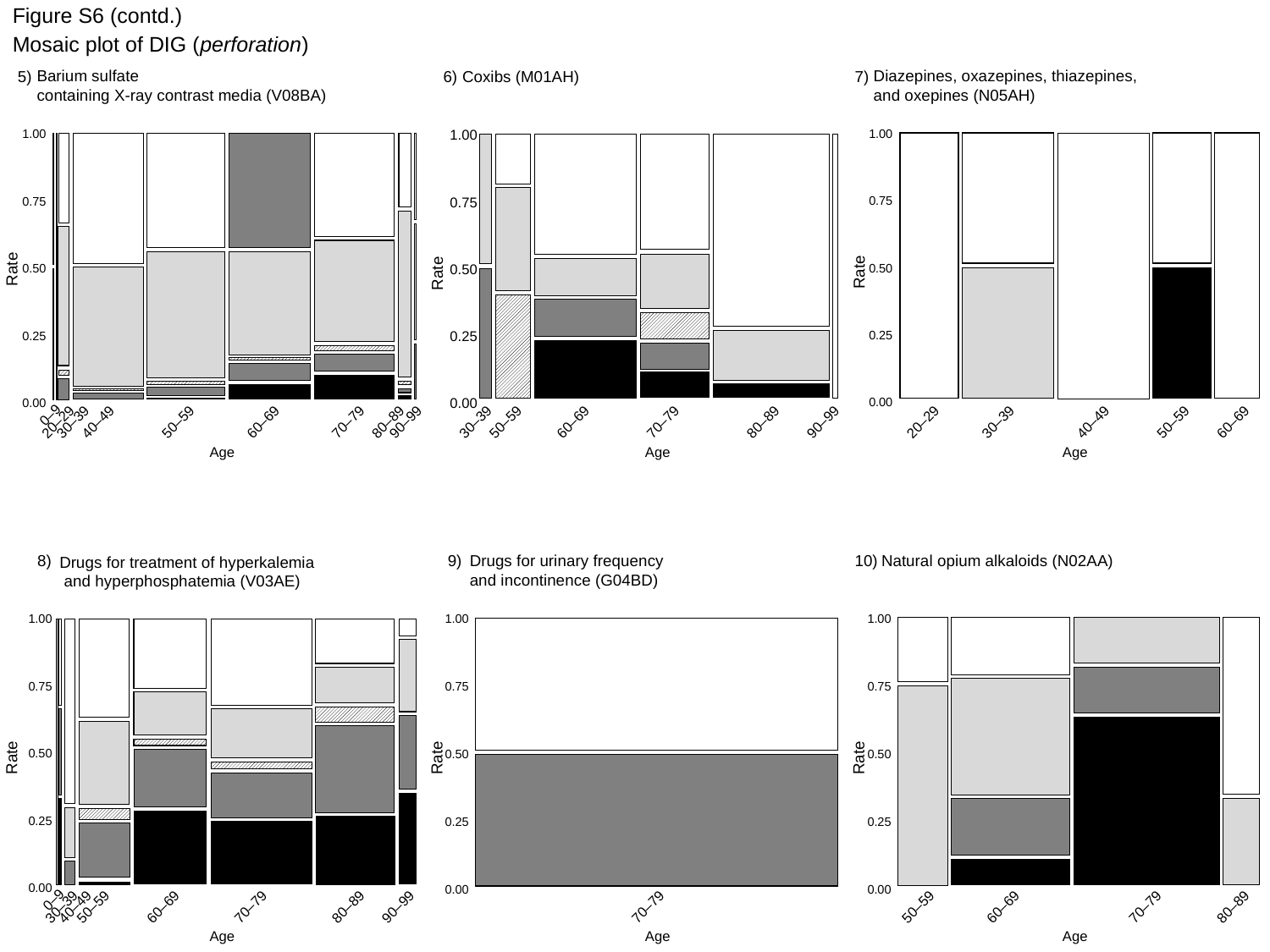

Figure S6 (contd.)
Mosaic plot of DIG (perforation)
5)
Barium sulfate
containing X-ray contrast media (V08BA)
6)
Coxibs (M01AH)
7)
Diazepines, oxazepines, thiazepines,
and oxepines (N05AH)
1.00
0.75
0.50
0.25
0.00
Rate
1.00
0.75
0.50
0.25
0.00
1.00
0.75
0.50
0.25
0.00
v
Rate
Rate
0–9
20–29
30–39
40–49
50–59
60–69
70–79
80–89
90–99
30–39
50–59
60–69
70–79
80–89
90–99
20–29
30–39
40–49
50–59
60–69
Age
Age
Age
 8)
Drugs for treatment of hyperkalemia
 and hyperphosphatemia (V03AE)
 9)
Drugs for urinary frequency
and incontinence (G04BD)
10)
Natural opium alkaloids (N02AA)
1.00
0.75
0.50
0.25
0.00
1.00
0.75
0.50
0.25
0.00
1.00
0.75
0.50
0.25
0.00
Rate
Rate
Rate
0–9
30–39
40–49
50–59
60–69
70–79
80–89
90–99
70–79
50–59
60–69
70–79
80–89
Age
Age
Age

## Slide 3
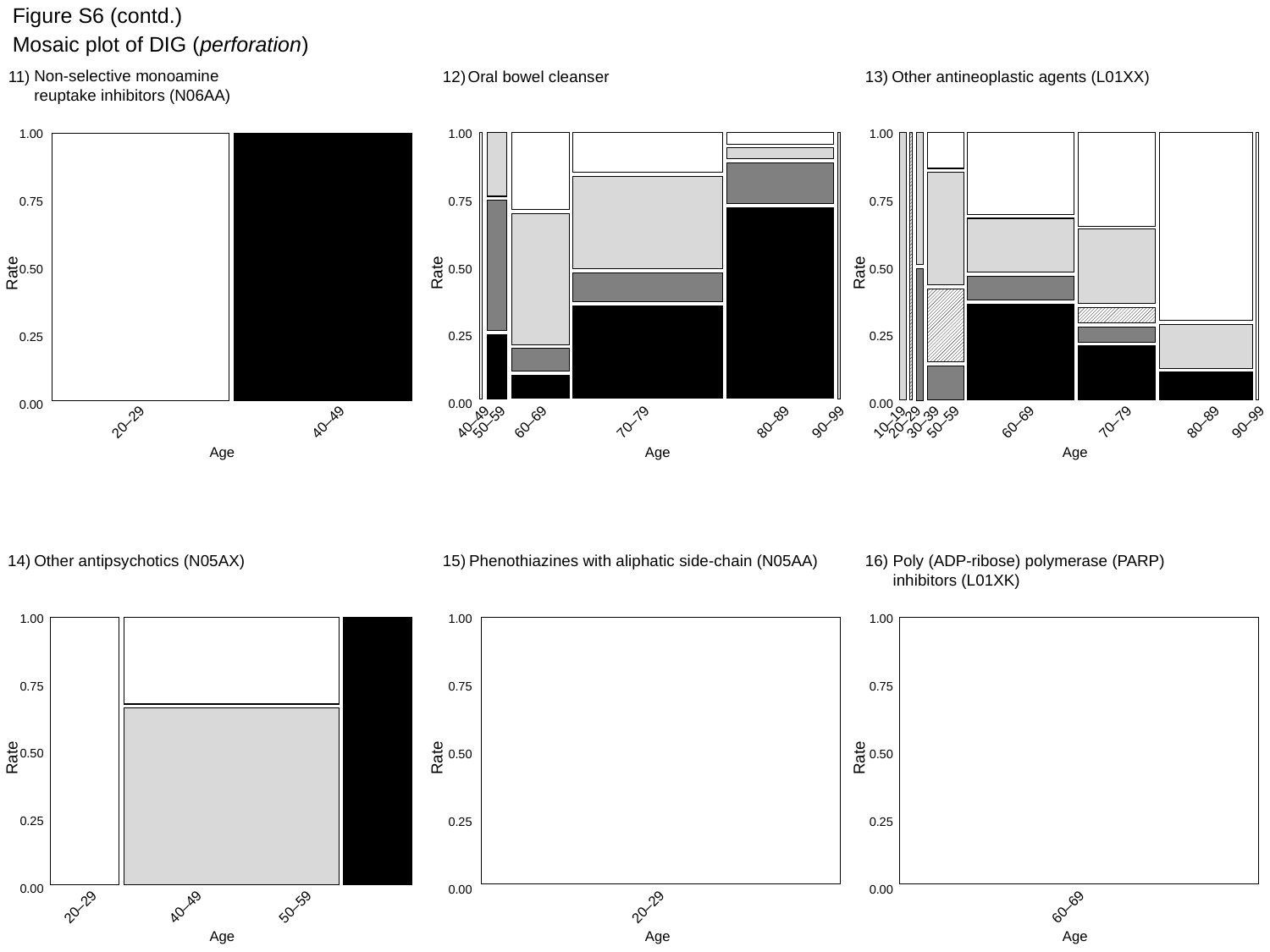

Figure S6 (contd.)
Mosaic plot of DIG (perforation)
11)
Non-selective monoamine
reuptake inhibitors (N06AA)
12)
Oral bowel cleanser
13)
Other antineoplastic agents (L01XX)
1.00
0.75
0.50
0.25
0.00
1.00
0.75
0.50
0.25
0.00
1.00
0.75
0.50
0.25
0.00
Rate
Rate
Rate
20–29
40–49
40–49
50–59
60–69
70–79
80–89
90–99
10–19
20–29
30–39
50–59
60–69
70–79
80–89
90–99
Age
Age
Age
14)
Other antipsychotics (N05AX)
15)
Phenothiazines with aliphatic side-chain (N05AA)
16)
Poly (ADP-ribose) polymerase (PARP)
inhibitors (L01XK)
1.00
0.75
0.50
0.25
0.00
1.00
0.75
0.50
0.25
0.00
1.00
0.75
0.50
0.25
0.00
Rate
Rate
Rate
20–29
40–49
50–59
20–29
60–69
Age
Age
Age

## Slide 4
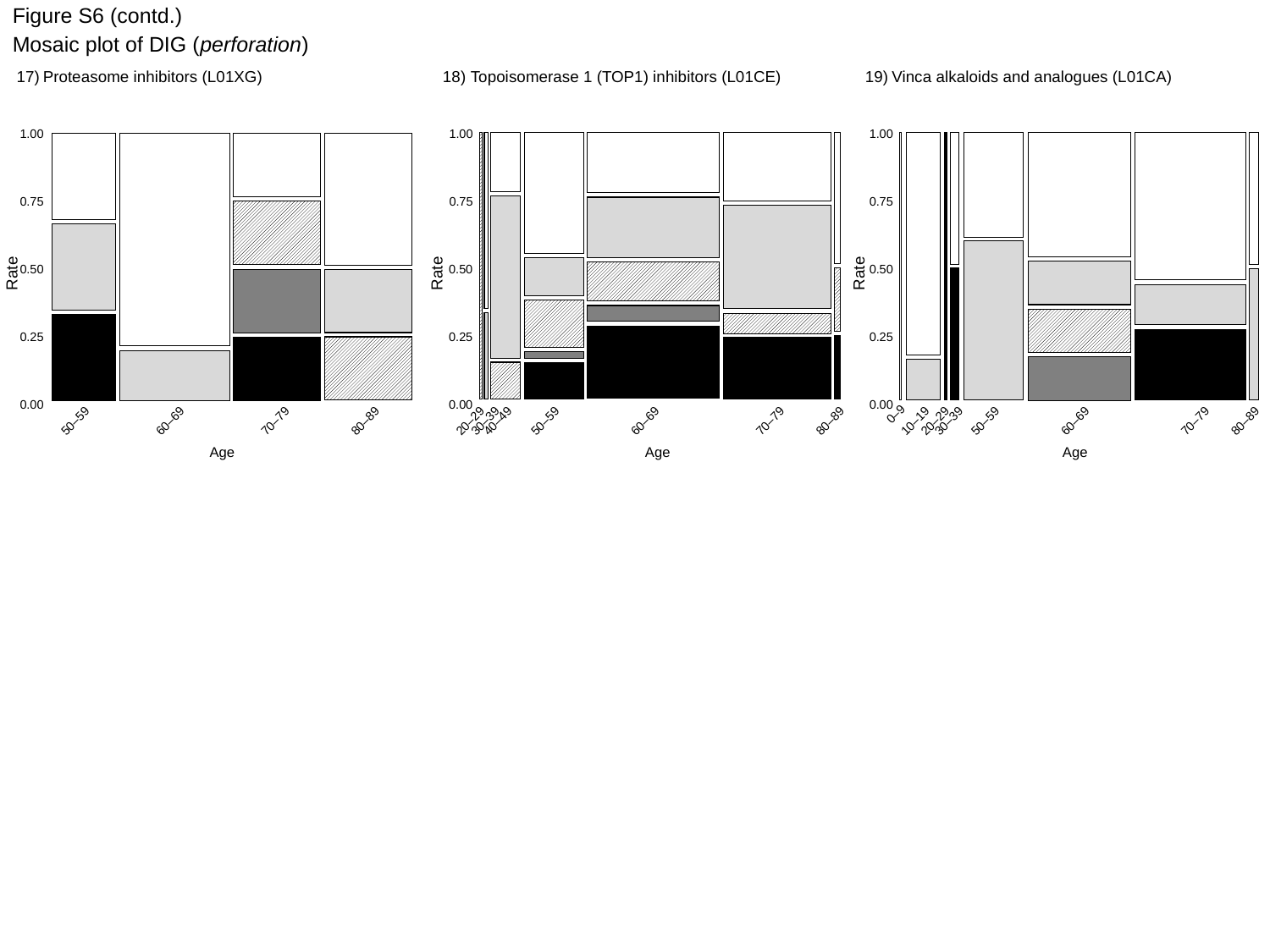

Figure S6 (contd.)
Mosaic plot of DIG (perforation)
17)
Proteasome inhibitors (L01XG)
18)
Topoisomerase 1 (TOP1) inhibitors (L01CE)
19)
Vinca alkaloids and analogues (L01CA)
1.00
0.75
0.50
0.25
0.00
1.00
0.75
0.50
0.25
0.00
1.00
0.75
0.50
0.25
0.00
Rate
Rate
Rate
0–9
50–59
60–69
70–79
80–89
20–29
30–39
40–49
50–59
60–69
70–79
80–89
10–19
20–29
30–39
50–59
60–69
70–79
80–89
Age
Age
Age
