# Supplementary material for: Analysis of Drug-Induced Gastrointestinal Obstruction and Perforation Using the Japanese Adverse Drug Event Report Database
Source: Front Pharmacol. 2021 Jul 26;12:692292. doi: 10.3389/fphar.2021.692292 (PMC8350341; doi:10.3389/fphar.2021.692292)
Supplement: Supplementary file 6 [file Presentation3.PPTX]

## Slide 1
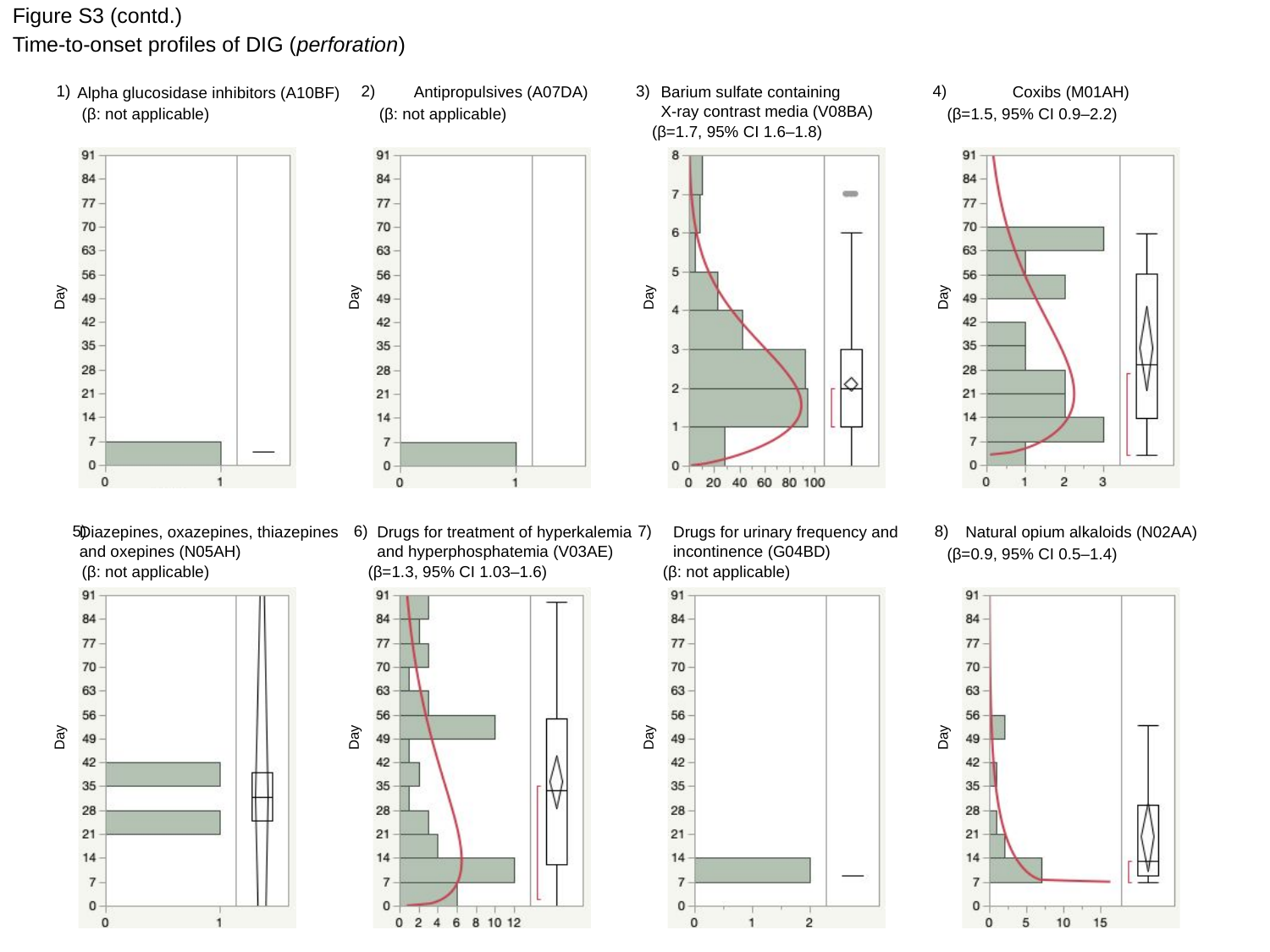

Figure S3 (contd.)
Time-to-onset profiles of DIG (perforation)
1)
2)
3)
4)
Antipropulsives (A07DA)
Coxibs (M01AH)
Alpha glucosidase inhibitors (A10BF)
Barium sulfate containing
X-ray contrast media (V08BA)
(β=1.5, 95% CI 0.9–2.2)
(β=1.7, 95% CI 1.6–1.8)
Day
Day
Day
Day
5)
6)
7)
8)
Diazepines, oxazepines, thiazepines
and oxepines (N05AH)
Drugs for treatment of hyperkalemia
and hyperphosphatemia (V03AE)
Drugs for urinary frequency and
incontinence (G04BD)
Natural opium alkaloids (N02AA)
(β=0.9, 95% CI 0.5–1.4)
(β=1.3, 95% CI 1.03–1.6)
Day
Day
Day
Day
(β: not applicable)
(β: not applicable)
(β: not applicable)
(β: not applicable)

## Slide 2
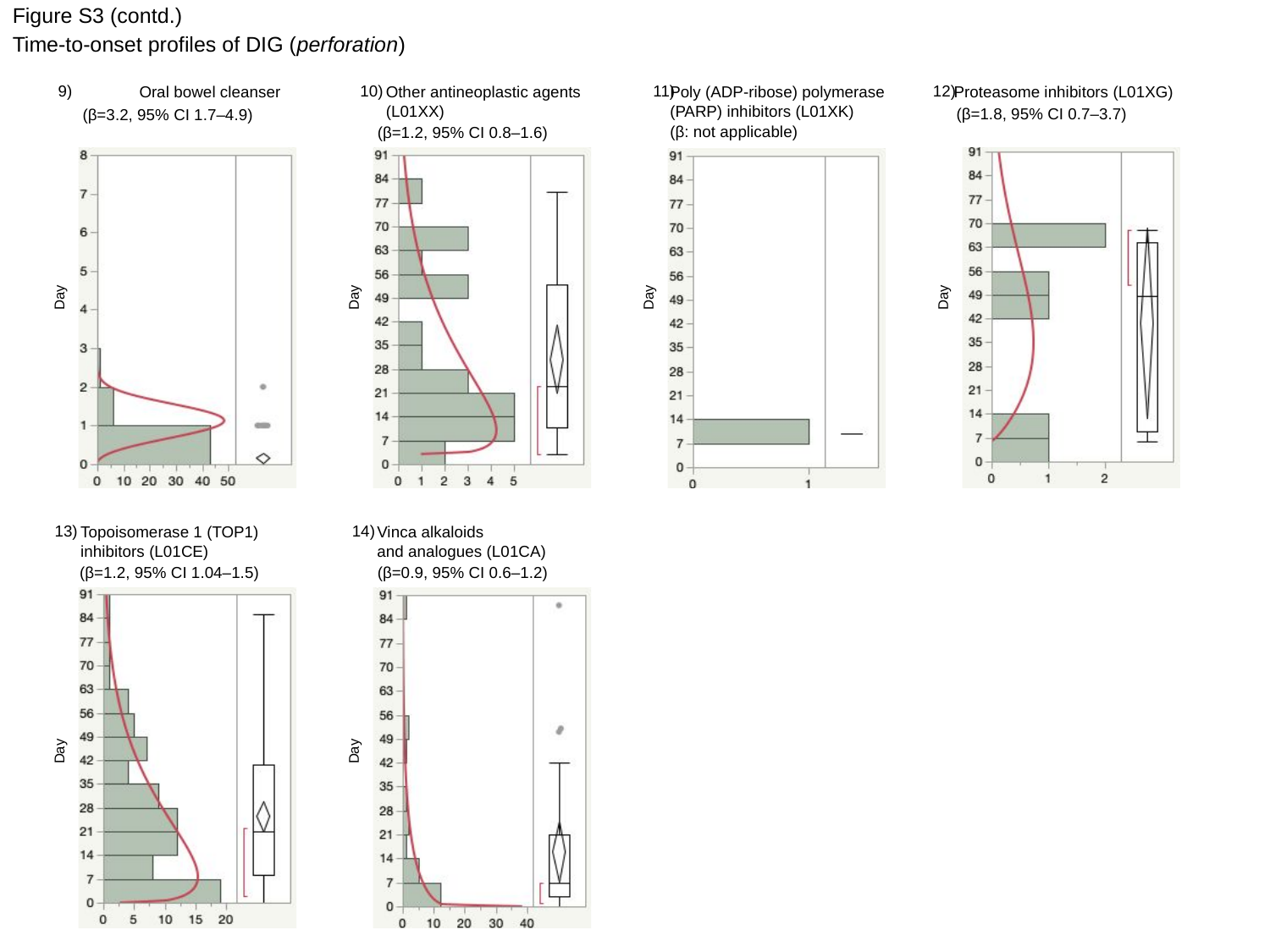

Figure S3 (contd.)
Time-to-onset profiles of DIG (perforation)
9)
10)
11)
12)
Oral bowel cleanser
Other antineoplastic agents
(L01XX)
Poly (ADP-ribose) polymerase
(PARP) inhibitors (L01XK)
Proteasome inhibitors (L01XG)
(β=1.8, 95% CI 0.7–3.7)
(β=3.2, 95% CI 1.7–4.9)
(β=1.2, 95% CI 0.8–1.6)
Day
Day
Day
Day
13)
14)
Topoisomerase 1 (TOP1)
inhibitors (L01CE)
Vinca alkaloids
and analogues (L01CA)
(β=1.2, 95% CI 1.04–1.5)
(β=0.9, 95% CI 0.6–1.2)
Day
Day
(β: not applicable)
