# Supplementary material for: Analysis of Drug-Induced Gastrointestinal Obstruction and Perforation Using the Japanese Adverse Drug Event Report Database
Source: Front Pharmacol. 2021 Jul 26;12:692292. doi: 10.3389/fphar.2021.692292 (PMC8350341; doi:10.3389/fphar.2021.692292)
Supplement: Supplementary file 7 [file Presentation2.PPTX]

## Slide 1
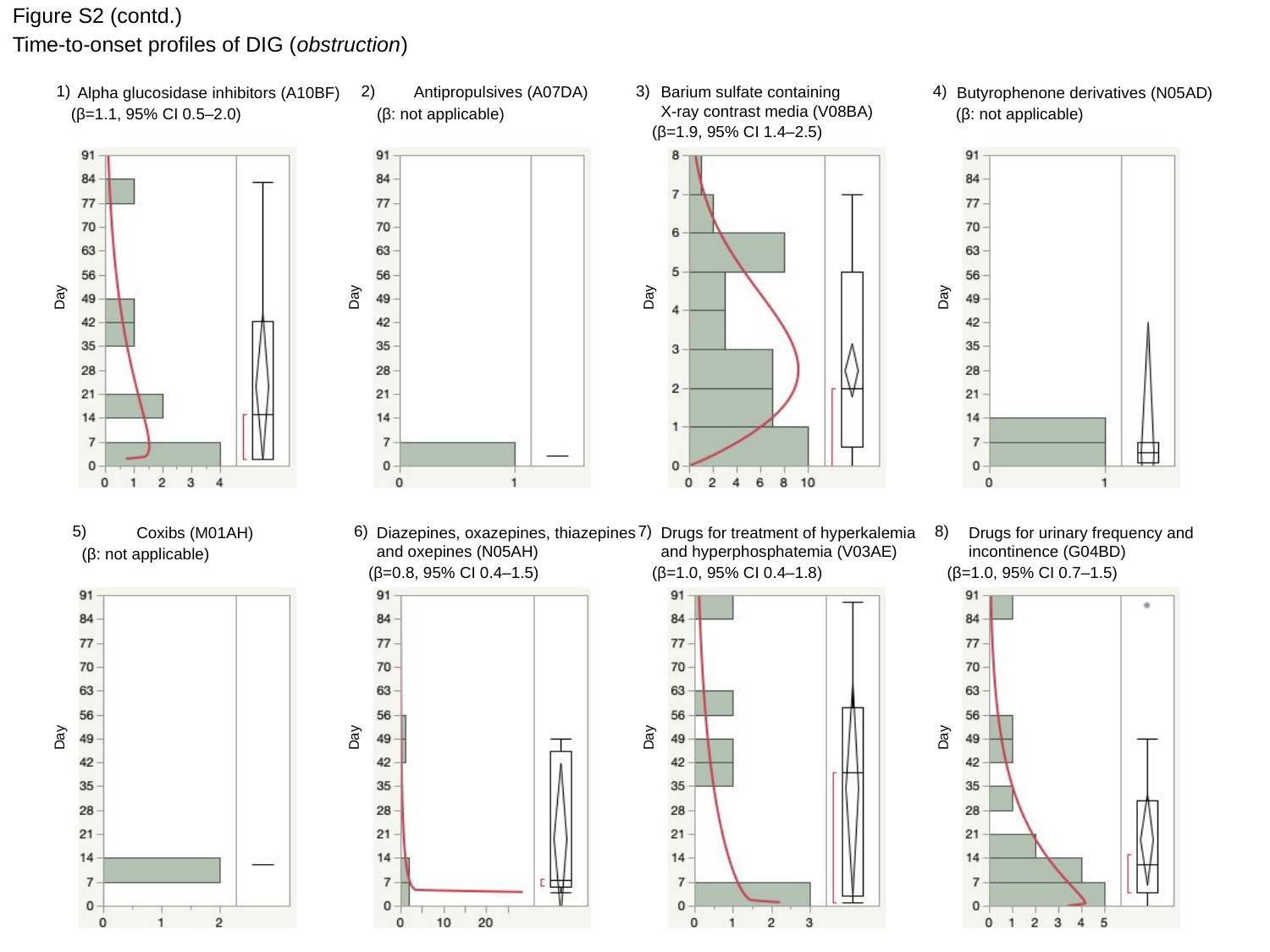

Figure S2 (contd.)
Time-to-onset profiles of DIG (obstruction)
1)
Alpha glucosidase inhibitors (A10BF)
2)
Antipropulsives (A07DA)
3)
Barium sulfate containing
X-ray contrast media (V08BA)
4)
Butyrophenone derivatives (N05AD)
(β=1.1, 95% CI 0.5–2.0)
(β=1.9, 95% CI 1.4–2.5)
Day
Day
Day
Day
5)
Coxibs (M01AH)
6)
Diazepines, oxazepines, thiazepines
and oxepines (N05AH)
7)
Drugs for treatment of hyperkalemia
and hyperphosphatemia (V03AE)
8)
Drugs for urinary frequency and
incontinence (G04BD)
(β=0.8, 95% CI 0.4–1.5)
(β=1.0, 95% CI 0.4–1.8)
(β=1.0, 95% CI 0.7–1.5)
Day
Day
Day
Day
(β: not applicable)
(β: not applicable)
(β: not applicable)

## Slide 2
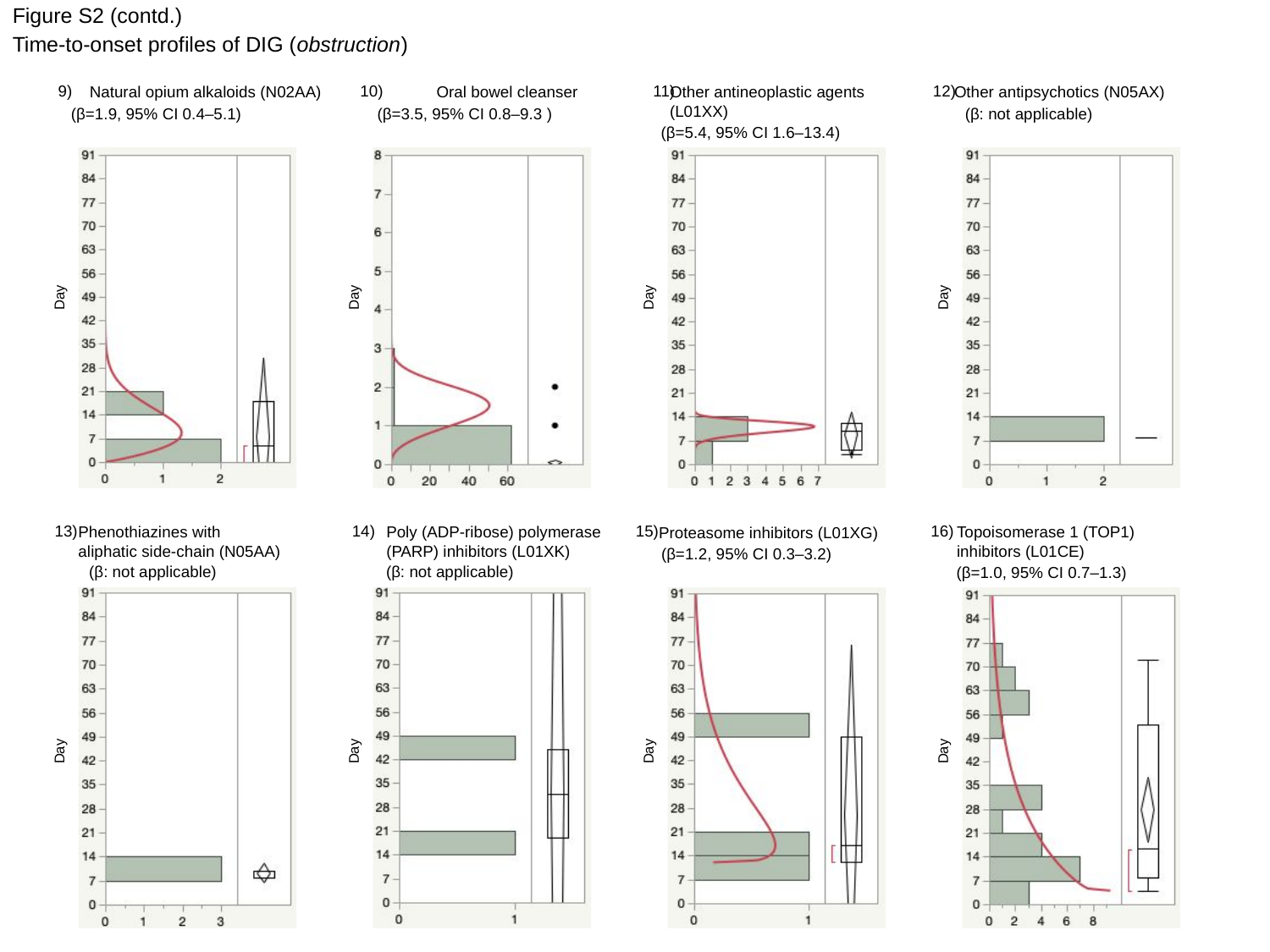

Figure S2 (contd.)
Time-to-onset profiles of DIG (obstruction)
9)
10)
11)
12)
Natural opium alkaloids (N02AA)
Oral bowel cleanser
Other antineoplastic agents
(L01XX)
Other antipsychotics (N05AX)
(β=1.9, 95% CI 0.4–5.1)
(β=3.5, 95% CI 0.8–9.3 )
(β=5.4, 95% CI 1.6–13.4)
Day
Day
Day
Day
13)
14)
15)
16)
Phenothiazines with
aliphatic side-chain (N05AA)
Poly (ADP-ribose) polymerase
(PARP) inhibitors (L01XK)
Proteasome inhibitors (L01XG)
Topoisomerase 1 (TOP1)
inhibitors (L01CE)
(β=1.2, 95% CI 0.3–3.2)
(β=1.0, 95% CI 0.7–1.3)
Day
Day
Day
Day
(β: not applicable)
(β: not applicable)
(β: not applicable)

## Slide 3
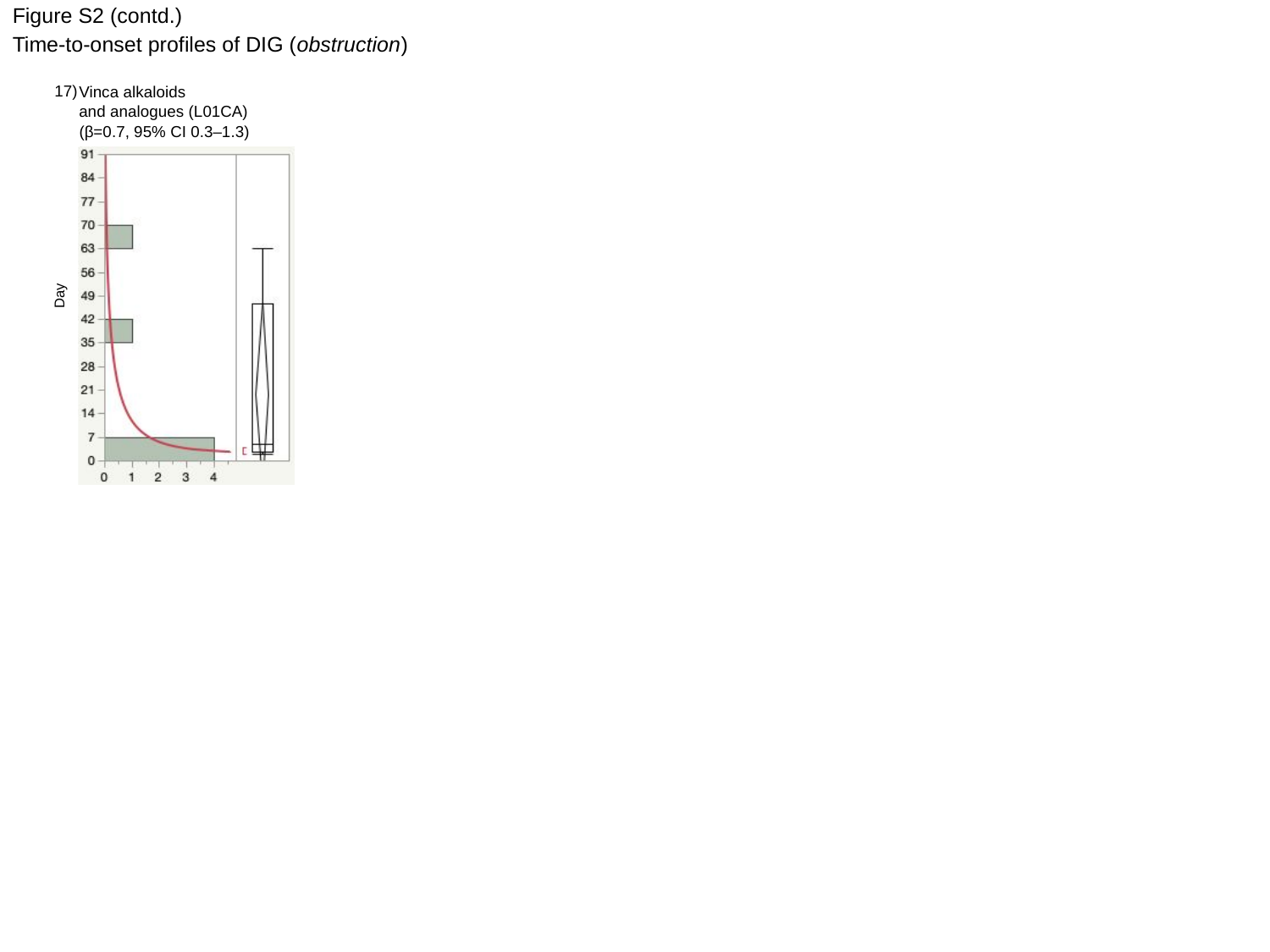

Figure S2 (contd.)
Time-to-onset profiles of DIG (obstruction)
17)
Vinca alkaloids
and analogues (L01CA)
(β=0.7, 95% CI 0.3–1.3)
Day
