# Supplementary material for: Analysis of Drug-Induced Gastrointestinal Obstruction and Perforation Using the Japanese Adverse Drug Event Report Database
Source: Front Pharmacol. 2021 Jul 26;12:692292. doi: 10.3389/fphar.2021.692292 (PMC8350341; doi:10.3389/fphar.2021.692292)
Supplement: Supplementary file 8 [file Presentation7.PPTX]

## Slide 1
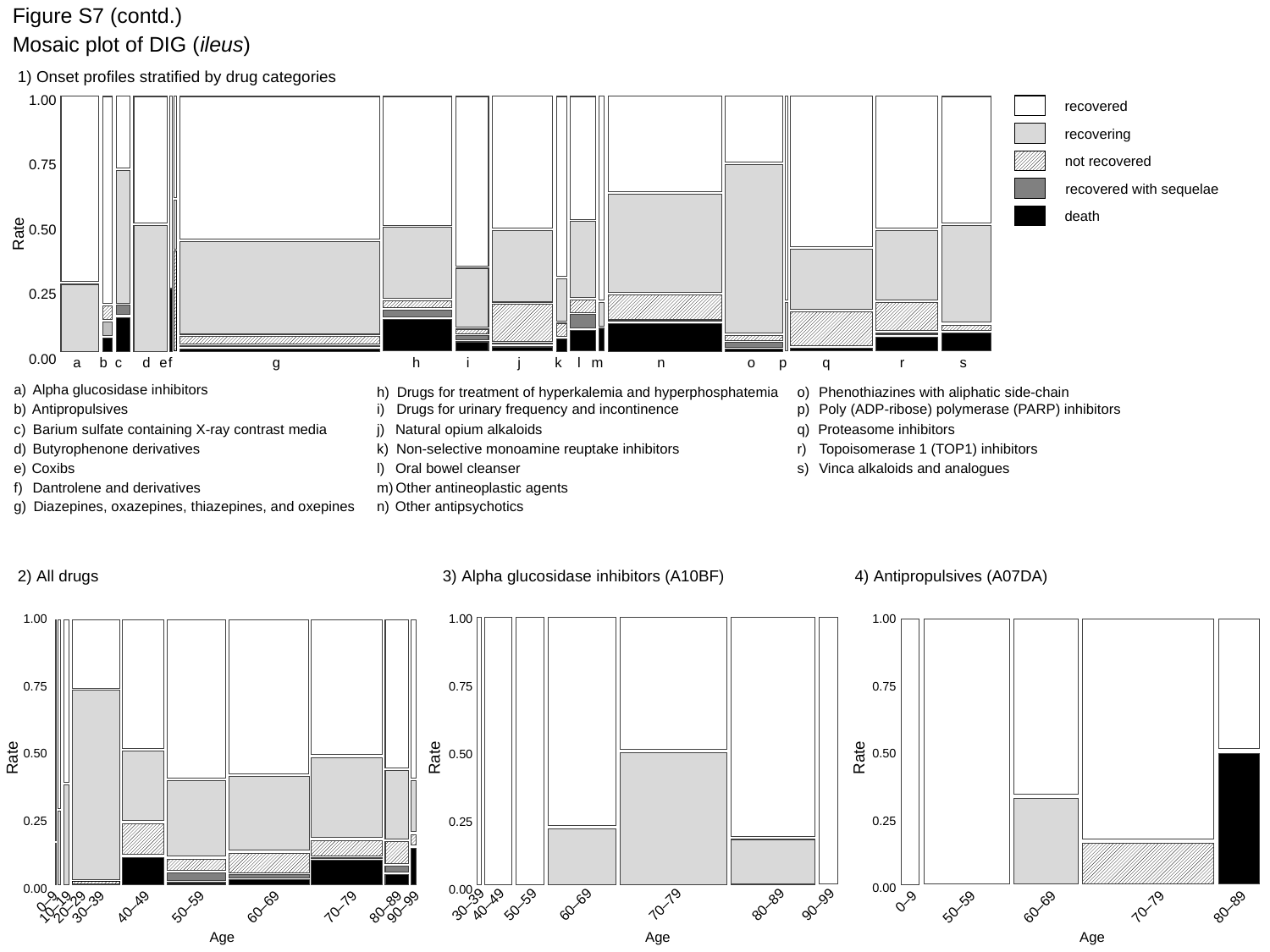

Figure S7 (contd.)
Mosaic plot of DIG (ileus)
1)
Onset profiles stratified by drug categories
1.00
0.75
0.50
0.25
0.00
recovered
recovering
not recovered
recovered with sequelae
death
Rate
a
b
c
d
e
f
g
h
i
j
k
l
m
n
o
p
q
r
s
a)
Alpha glucosidase inhibitors
h)
Drugs for treatment of hyperkalemia and hyperphosphatemia
o)
Phenothiazines with aliphatic side-chain
p)
Poly (ADP-ribose) polymerase (PARP) inhibitors
q)
Proteasome inhibitors
r)
Topoisomerase 1 (TOP1) inhibitors
s)
Vinca alkaloids and analogues
b)
Antipropulsives
i)
Drugs for urinary frequency and incontinence
c)
Barium sulfate containing X-ray contrast media
j)
Natural opium alkaloids
d)
Butyrophenone derivatives
k)
Non-selective monoamine reuptake inhibitors
e)
Coxibs
l)
Oral bowel cleanser
f)
Dantrolene and derivatives
m)
Other antineoplastic agents
g)
Diazepines, oxazepines, thiazepines, and oxepines
n)
Other antipsychotics
2)
All drugs
3)
Alpha glucosidase inhibitors (A10BF)
4)
Antipropulsives (A07DA)
1.00
0.75
0.50
0.25
0.00
1.00
0.75
0.50
0.25
0.00
1.00
0.75
0.50
0.25
0.00
Rate
Rate
Rate
0–9
0–9
50–59
90–99
30–39
40–49
60–69
70–79
80–89
10–19
20–29
30–39
40–49
50–59
60–69
70–79
80–89
90–99
50–59
60–69
70–79
80–89
Age
Age
Age

## Slide 2
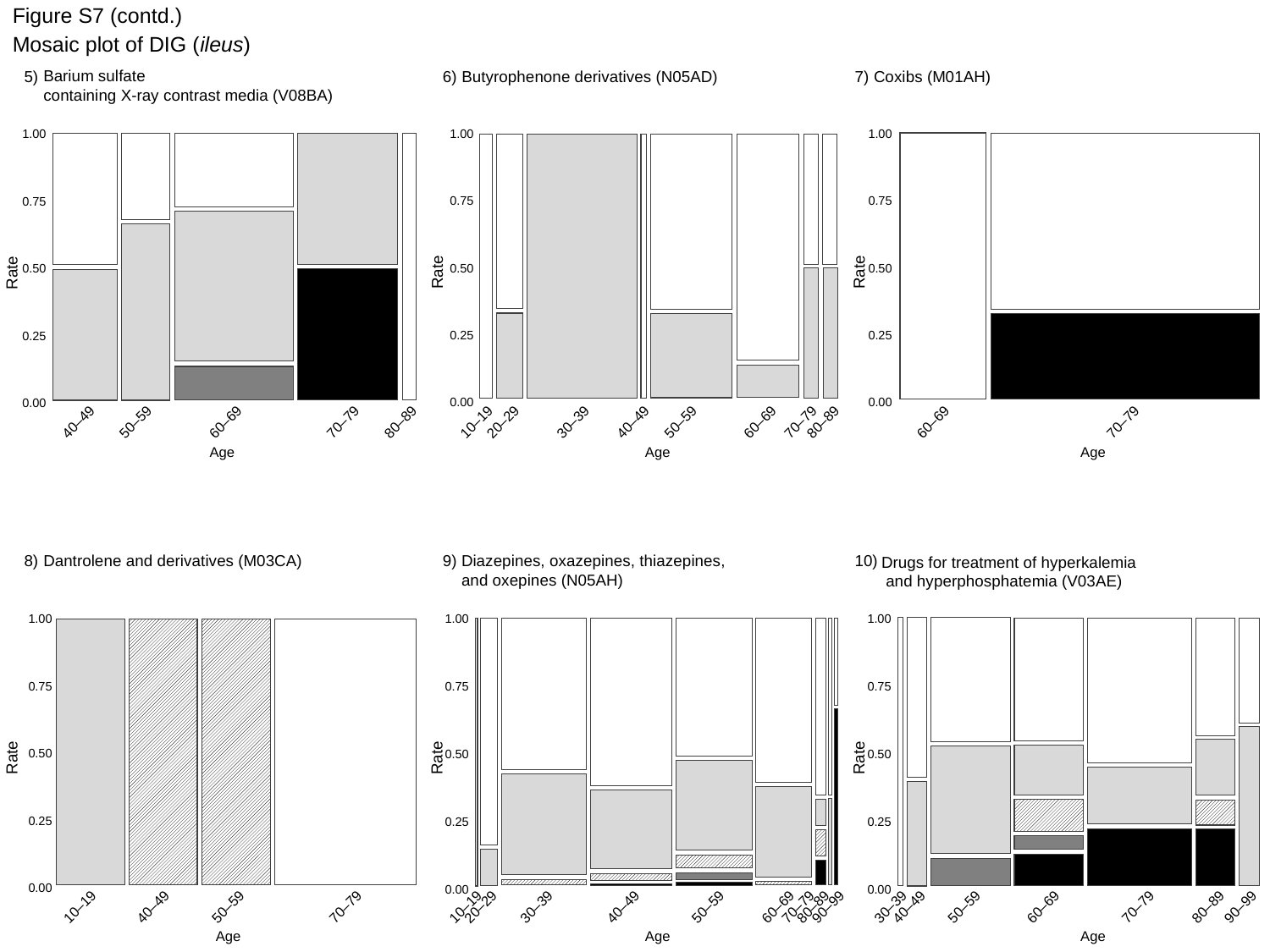

Figure S7 (contd.)
Mosaic plot of DIG (ileus)
5)
Barium sulfate
containing X-ray contrast media (V08BA)
6)
Butyrophenone derivatives (N05AD)
7)
Coxibs (M01AH)
1.00
0.75
0.50
0.25
0.00
1.00
0.75
0.50
0.25
0.00
1.00
0.75
0.50
0.25
0.00
Rate
Rate
Rate
40–49
50–59
60–69
70–79
80–89
10–19
20–29
30–39
40–49
50–59
60–69
70–79
80–89
60–69
70–79
Age
Age
Age
8)
Dantrolene and derivatives (M03CA)
9)
Diazepines, oxazepines, thiazepines,
and oxepines (N05AH)
10)
Drugs for treatment of hyperkalemia
 and hyperphosphatemia (V03AE)
1.00
0.75
0.50
0.25
0.00
1.00
0.75
0.50
0.25
0.00
1.00
0.75
0.50
0.25
0.00
1
Rate
Rate
Rate
10–19
40–49
50–59
70–79
10–19
20–29
30–39
40–49
50–59
60–69
70–79
80–89
90–99
30–39
40–49
50–59
60–69
70–79
80–89
90–99
Age
Age
Age

## Slide 3
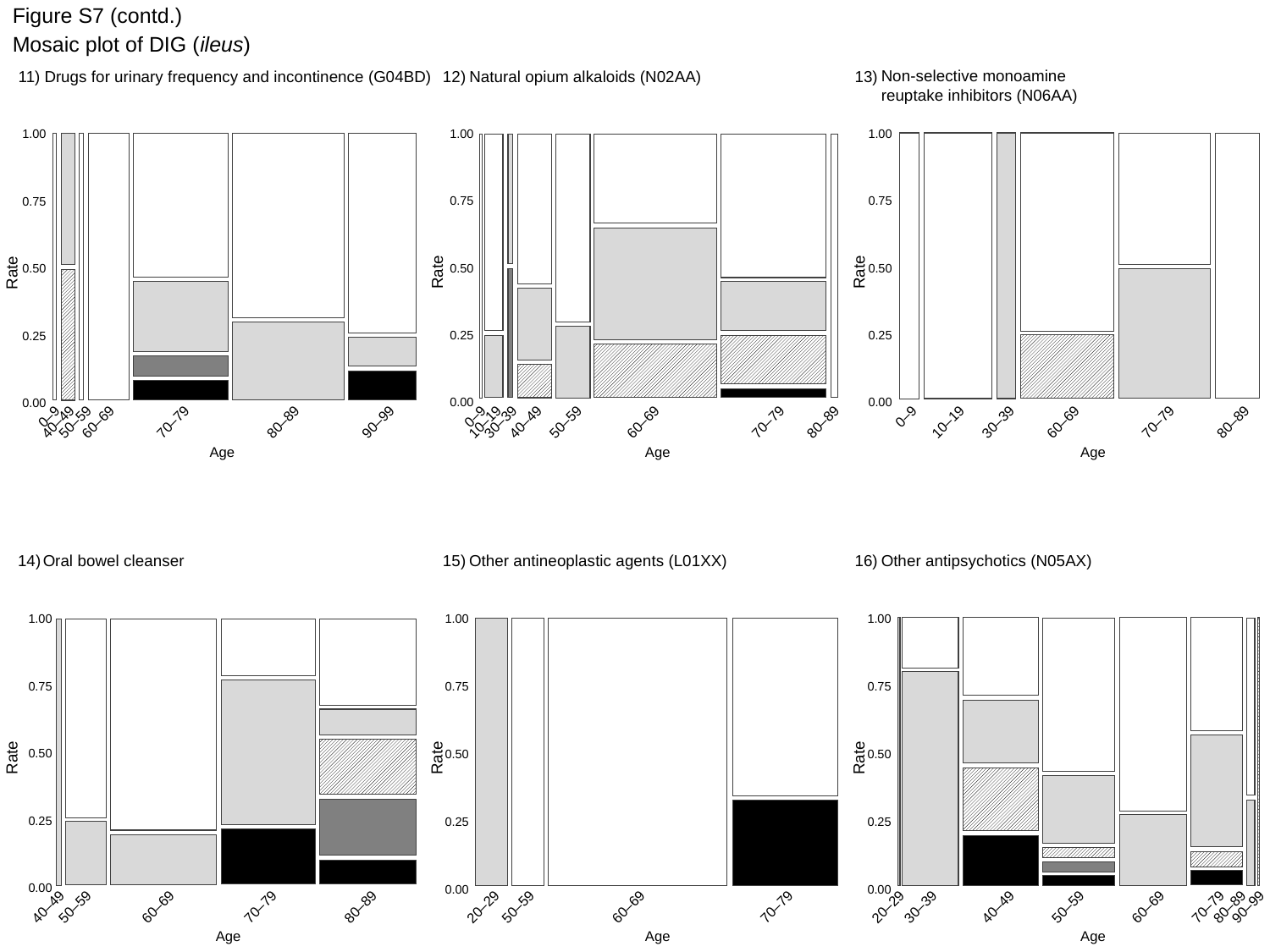

Figure S7 (contd.)
Mosaic plot of DIG (ileus)
11)
Drugs for urinary frequency and incontinence (G04BD)
12)
Natural opium alkaloids (N02AA)
13)
Non-selective monoamine
reuptake inhibitors (N06AA)
1.00
0.75
0.50
0.25
0.00
1.00
0.75
0.50
0.25
0.00
1.00
0.75
0.50
0.25
0.00
Rate
Rate
Rate
0–9
0–9
0–9
40–49
50–59
60–69
70–79
80–89
90–99
10–19
30–39
40–49
50–59
60–69
70–79
80–89
10–19
30–39
60–69
70–79
80–89
Age
Age
Age
14)
Oral bowel cleanser
15)
Other antineoplastic agents (L01XX)
16)
Other antipsychotics (N05AX)
1.00
0.75
0.50
0.25
0.00
1.00
0.75
0.50
0.25
0.00
1.00
0.75
0.50
0.25
0.00
Rate
Rate
Rate
40–49
50–59
60–69
70–79
80–89
20–29
50–59
60–69
70–79
20–29
30–39
40–49
50–59
60–69
70–79
80–89
90–99
Age
Age
Age

## Slide 4
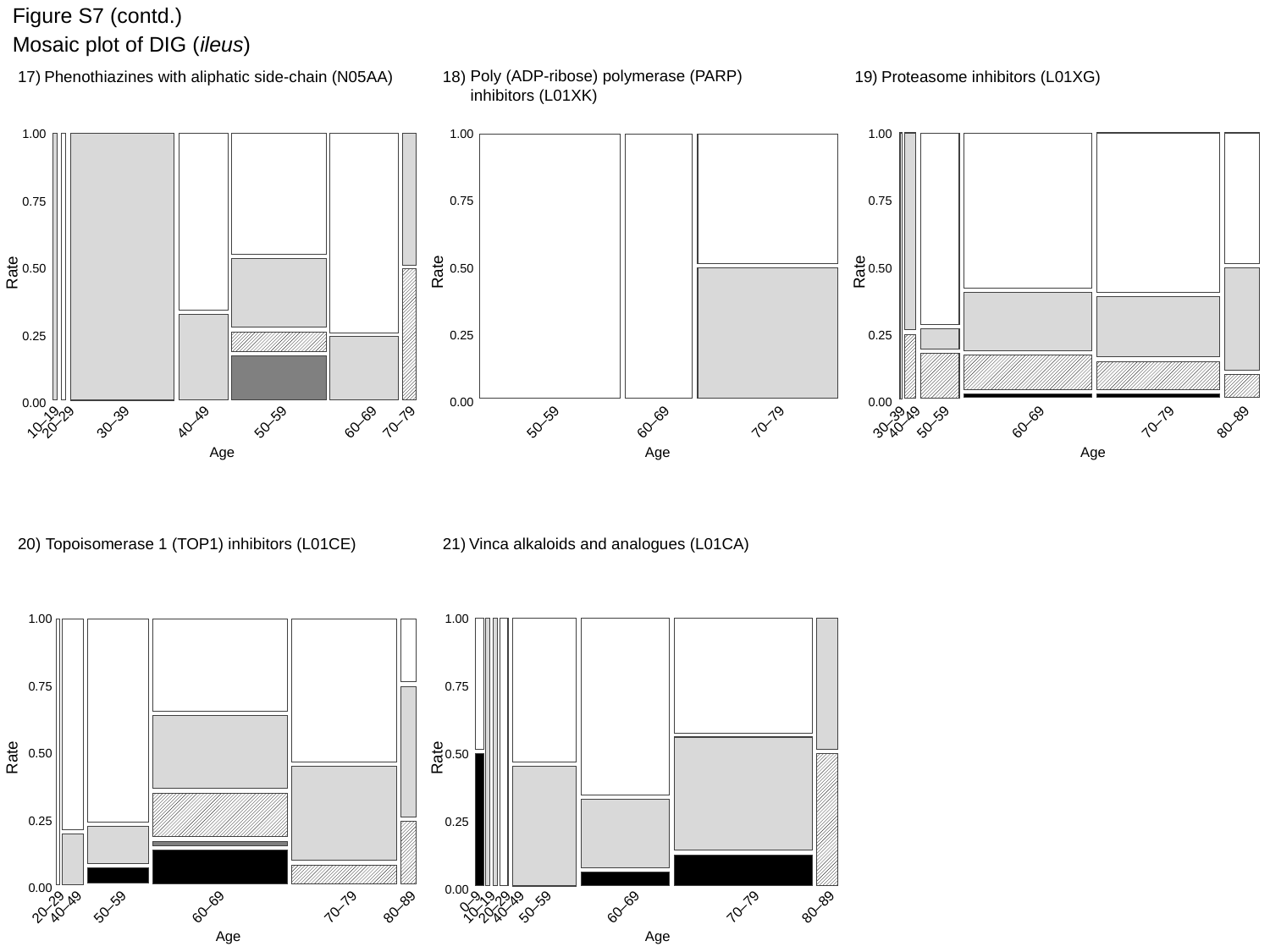

Figure S7 (contd.)
Mosaic plot of DIG (ileus)
17)
Phenothiazines with aliphatic side-chain (N05AA)
18)
Poly (ADP-ribose) polymerase (PARP)
inhibitors (L01XK)
19)
Proteasome inhibitors (L01XG)
1.00
0.75
0.50
0.25
0.00
1.00
0.75
0.50
0.25
0.00
1.00
0.75
0.50
0.25
0.00
Rate
Rate
Rate
50–59
60–69
70–79
10–19
20–29
30–39
40–49
50–59
60–69
70–79
30–39
40–49
50–59
60–69
70–79
80–89
Age
Age
Age
20)
Topoisomerase 1 (TOP1) inhibitors (L01CE)
21)
Vinca alkaloids and analogues (L01CA)
1.00
0.75
0.50
0.25
0.00
1.00
0.75
0.50
0.25
0.00
Rate
Rate
0–9
20–29
40–49
50–59
60–69
70–79
80–89
10–19
20–29
40–49
50–59
60–69
70–79
80–89
Age
Age
